# Supplementary material for: Dehydroamino acids and their crosslinks in Alzheimer’s disease aggregates
Source: Brain Commun. 2025 Jan 16;7(1):fcaf019. doi: 10.1093/braincomms/fcaf019 (PMC11775630; doi:10.1093/braincomms/fcaf019)
Supplement: fcaf019_Supplementary_Data [file fcaf019_supplementary_data.zip › Supplementary Material.pdf]

This Supplementary Material 1 file includes:

Supplementary Text 1-3

Supplementary Figures 1-14

Supplementary Tables 1, 2, 5, and 6

Supplementary Schemes 1 and 2

Other Supplementary Materials for this manuscript include the following:

Supplementary Data 1 and 2

Supplementary Tables 3, 4, 7, 8

Supplementary Text 1

We chose to search for DHAAs and five DHAA conjugates: GSH-DHAA, HC-DHAA, DTT-DHAA, TCEP-DHAA, and DHAA-XLs. The DHAA-XLs were searched for separately in crosslink searches and then added as modifications on particular residues, as described in Methods. DTT and TCEP are both nucleophiles added as reducing agents (DTT in samples prepared by Wesseling *et. al.*[1] and TCEP in samples prepared by our laboratory). We chose to include two biological small molecules, glutathione and homocysteine, as possible nucleophiles that may react with DHAAs, though several other biological nucleophiles exist in the human brain. We sought to limit our search space by limiting the number of conjugates, ultimately resulting in a better false discovery rate. We prioritized the most abundant and most nucleophilic molecules. Glutathione is the most abundant small molecule thiol in human cells.[2] It was also recently reported that the human protein LanCL catalyzes the addition of glutathione to DHAAs,[3] therefore making it important to include in our search for DHAA conjugates. Homocysteine was included because it is another high abundance thiol-based nucleophile. There is also evidence that homocysteine accumulates in the brain of patients with AD and may contribute to the pathogenesis.[4] There are several amine-based nucleophiles, including amino acids and biogenic amines such as dopamine, norepinephrine, epinephrine, histamine, and serotonin.[5] While these are nucleophilic and could react with DHAAs, they are less likely to react than glutathione or homocysteine due to the lower nucleophilicity of amines compared to thiols. The reactions of these other nucleophiles with DHAAs lies beyond the scope of this study but may be an area for further investigation.

Supplementary Text 2

While there are no therapeutics that target DHAAs directly, several therapeutic options are currently being tested for efficacy in AD that may inadvertently affect DHAA formation or its downstream reactivity. When DHAAs are enzymatically generated in microorganism proteins, they arise from phosphorylated precursors. Therefore, preventing the phosphorylation event at eliminylation sites may prevent DHAA formation. Several kinase inhibitors are currently under investigation for their efficacy in treating AD. These kinase inhibitors may target GSK,[7] Cdk5,[8] JNK3,[9] p38 MAPK,[9] and MEK1/2,[10] all of which are known to contribute to AD pathogenesis and phosphorylate Tau, where we find numerous eliminylation sites. Therefore, by preventing these kinases from acting on Tau, they may prevent DHAA formation. We note that a direct inhibitor cannot yet be designed for a hypothetical DHAA-generating enzyme, as there is no such enzyme known in humans to catalyze this reaction (though some are suspected).[11,12]

Another therapeutic option of note is glutathione (or similar derivatives), which are common antioxidants.[13] These supplements are meant to treat the detrimental oxidative stress observed in AD. While glutathione has many roles in the cell, the human enzyme LanCL is known to add glutathione to DHAAs. Thus, increasing glutathione concentrations by administering a related supplement may affect this pathway by increasing glutathionylation of DHAAs by LanCL, and thereby prevent downstream crosslinking or other potentially detrimental DHAA conjugations.

Supplementary Text 3

Analysis of the sarkosyl-insoluble material permitted the identification of DHAAs and their conjugates, including DHAA-XLs, in large aggregates (Tau neurofibrillary tangles). Soluble paired helical filament (PHF) Tau has been surmised to represent an early stage of PHF generation in AD (and subsequent NFT formation).[14–16] This soluble pathogenic Tau has also been proposed to be the most toxic Tau species.[17,18] Soluble PHF Tau was isolated by Wesseling *et al.*[1] in two steps. First, soluble protein was extracted using a sarkosyl-based lysis and ultracentrifugation to remove protein aggregates. Second, PHF Tau was isolated using immunoaffinity columns containing the MC-1 antibody, an antibody that specifically binds PHF Tau.[19] Enriched soluble PHF Tau was analyzed by bottom-up proteomics.[1]

In our searches of this PHF Tau data, we detect glutathionylated DHAAs at residues S262, S400, and S404 in Tau. We also detect the DHAA-mediated crosslink in Tau between S262-C291 using a crosslink-specific search and the FDR-controlled method we describe in our Methods section (see Figure 2). If this soluble PHF Tau is representative of early stages of Tau aggregation as others have posited,[14–16] then glutathionylated DHAAs and DHAA-mediated crosslinks are present in these early stages of aggregation.

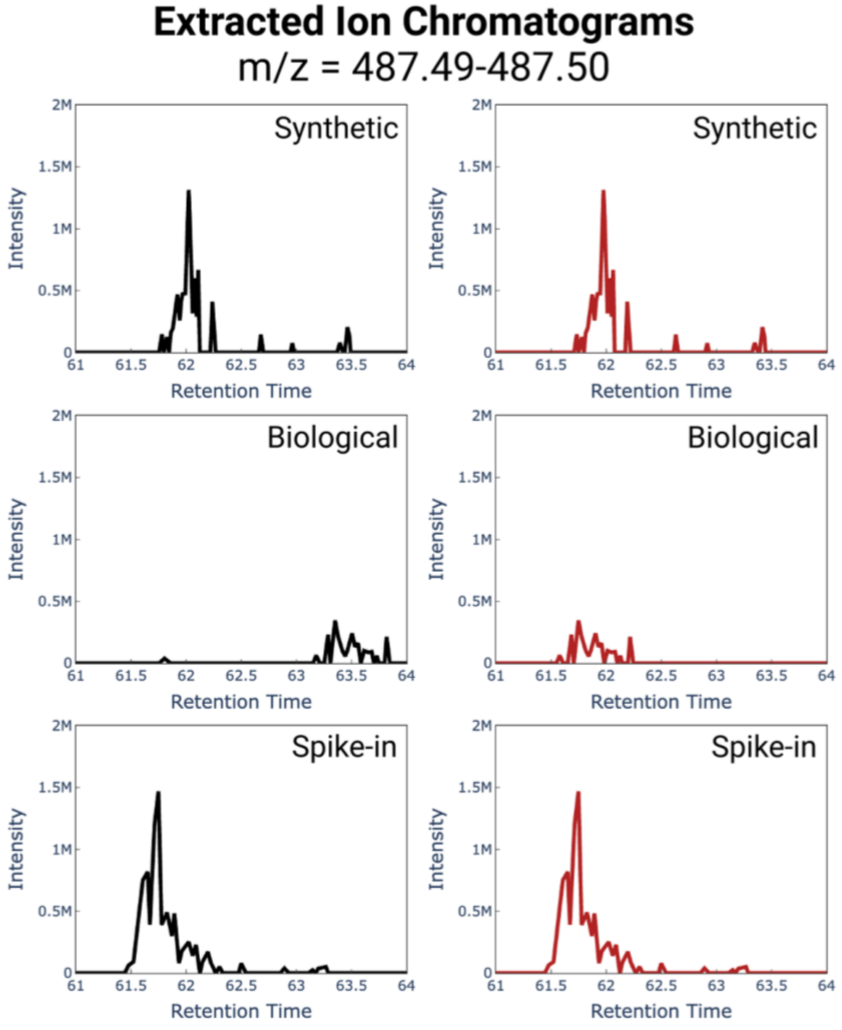

Supplementary Figure 1 Chromatographic evidence for a DHAA-mediated crosslink between residues S400-C291 in Tau. The extracted ion chromatograms (XIC) for the synthetic standard, the biological sample, and the spike-in are shown. The XIC displays chromatographic peaks for species detected with an m/z between 487.49 and 487.50. At z=4, the expected m/z of the crosslinked tryptic peptides is 487.495. On the left is raw data, shown in black, and on the right are the peaks for which retention times have been aligned. The synthetic standard (top panel) was spiked into a model human proteome (lysate from the immortalized human T-cell lymphocyte cell line, Jurkat) which served as both a carrier and to provide peptide references for retention time alignment.[58] Figure generated with BioRender.com and Plotly.[34]

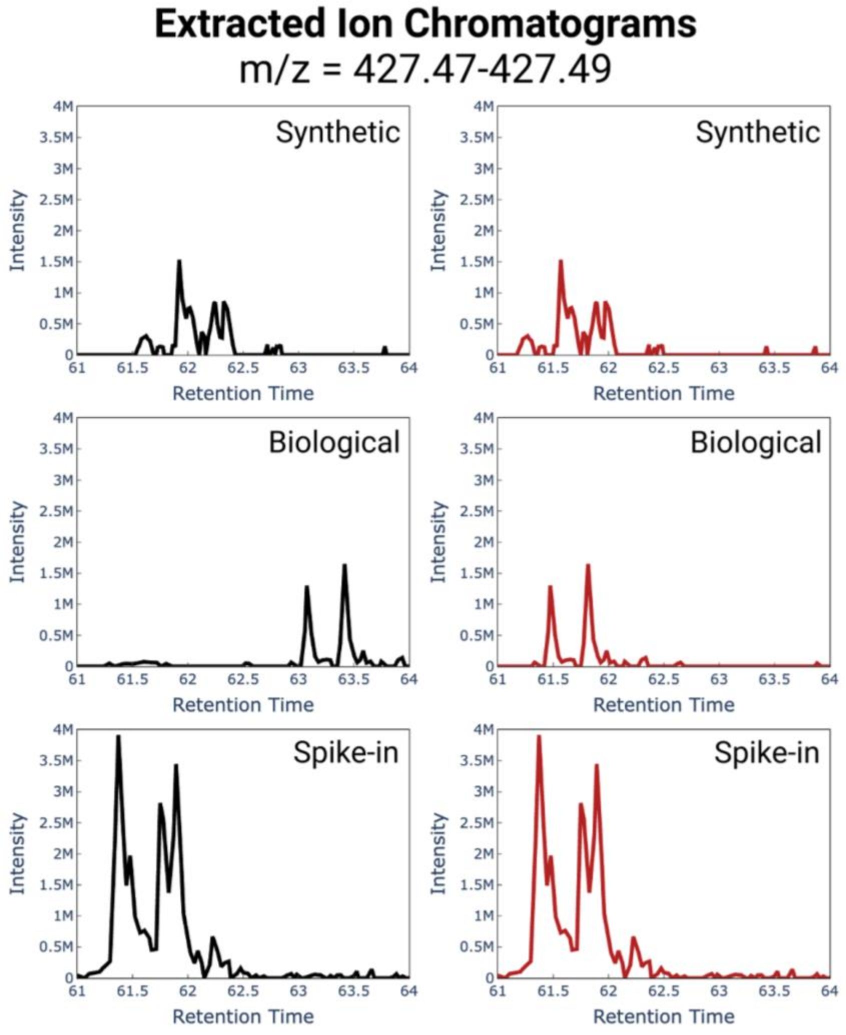

Supplementary Figure 2 Chromatographic evidence for a DHAA-mediated crosslink between residues S262-C291 in Tau. The extracted ion chromatograms (XIC) for the synthetic standard, the biological sample, and the spike-in are shown. The XIC displays chromatographic peaks for species detected with an m/z between 427.47 and 427.49. At z=4, the expected m/z of the crosslinked tryptic peptides is 427.474. On the left is raw data, shown in black, and on the right are the peaks for which retention times have been aligned. The synthetic standard (top panel) was spiked into a model human proteome (lysate from the immortalized human T-cell lymphocyte cell line, Jurkat) which served as both a carrier and to provide peptide references for retention time alignment.[58] Figure generated with BioRender.com and Plotly.[34]

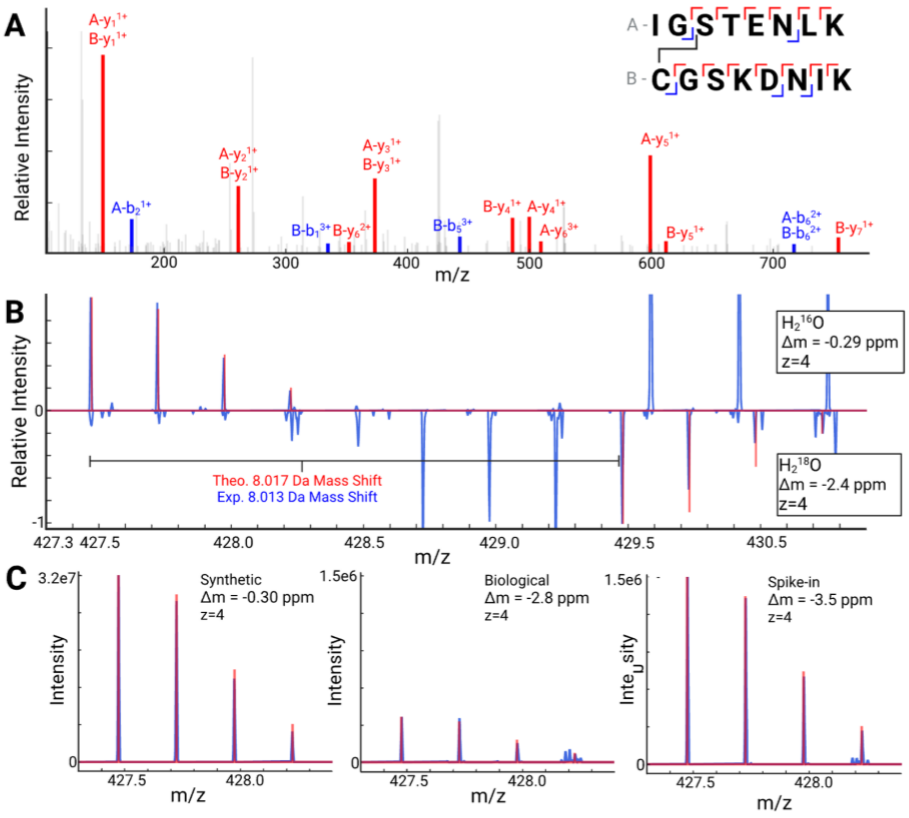

Supplementary Figure 3 Evidence for a DHAA-mediated crosslink between residues S262-C291 in Tau. **(A)** The MS2 fragmentation spectrum for the precursor mass corresponding to the crosslinked species, where m/z represents the mass to charge ratio. It shows thirteen y-ions and five b-ions, including five especially important fragments (A-y_6, A-b_6, B-b_1, B-b_5, and B-b_6) that include the crosslink. Fragmentation throughout the peptide is sufficient to accurately assign the crosslink to residues S262 and C291. **(B)** A mirror plot is shown with MS1 data derived from $H_2^{16}O$ samples on top (usual preparation) and $H_2^{18}O$ samples on the bottom (heavy isotope preparation). Raw data are shown in blue, and the theoretical isotope distributions are overlaid in red. The close match for each isotope peak compared to its theoretical value, for both mass and relative intensity, supports that the crosslink peptide has been identified correctly. Mass error is given in parts per million (ppm). Additional peaks in the $H_2^{16}O$ spectrum are from coeluting peptide species. Additional peaks in the $H_2^{18}O$ spectrum are in part from coeluting species, but also from incomplete isotopic labeling of the C-terminal oxygens. $H_2^{18}O$ labeling validates the presence of a crosslink between two peptides containing residues S262 and C291 in Tau. C.) MS1 spectra for the synthetic standard, the biological sample, and the spike-in are shown with

raw data in blue and theoretical isotope distributions overlaid in red. These data provide unequivocal evidence for a crosslink between S262 and C291 in Tau. Created with BioRender.com and Plotly.[34]

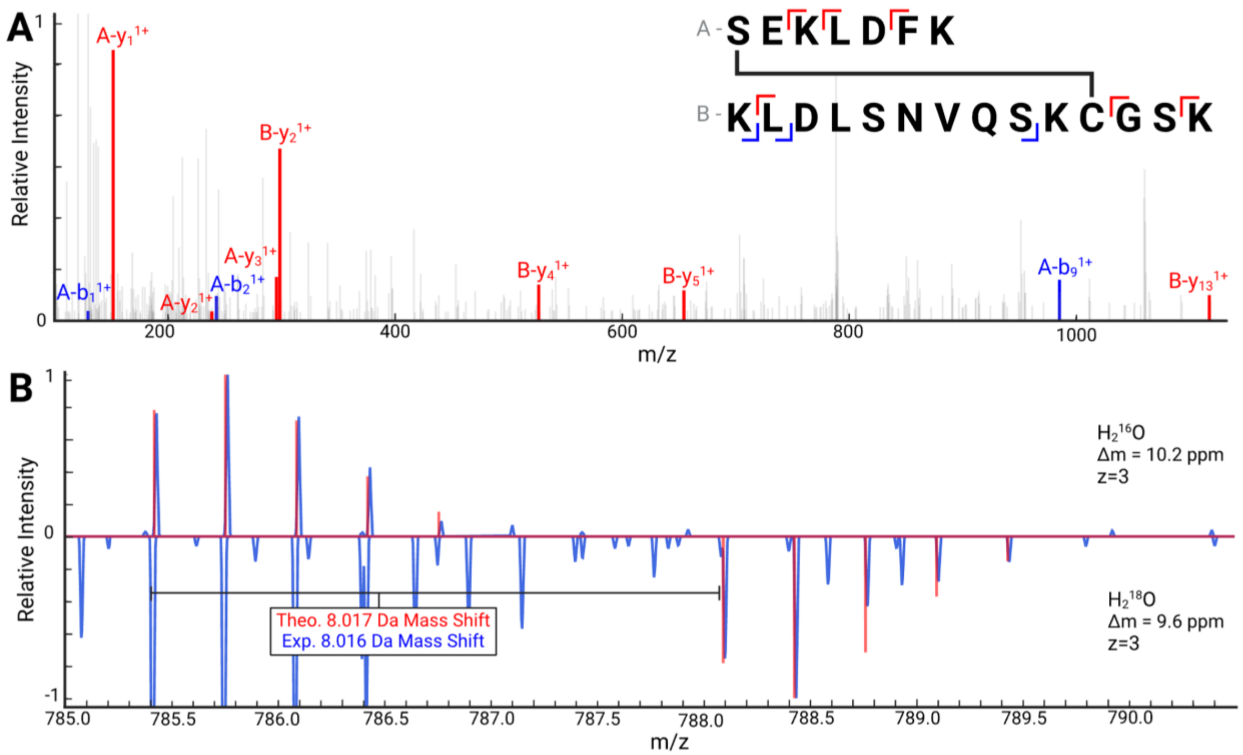

Supplementary Figure 4 Evidence for a DHAA-mediated crosslink between residues S341-C291 in Tau. **(A)** The MS2 fragmentation spectrum for the precursor mass corresponding to the crosslinked species, where m/z represents the mass to charge ratio. It shows six y-ions and three b-ions, including one especially important fragment (B-y_{13}) that includes the crosslink. Fragmentation throughout the peptide is sufficient to accurately assign the crosslink to S341 but is ambiguous between K290 and C291. Other spectra localize the crosslink to C291 (data not shown). **(B)** A mirror plot is shown with MS1 data derived from $H_2{}^{16}O$ samples on top (usual preparation) and $H_2{}^{18}O$ samples on the bottom (heavy isotope preparation). Raw data are shown in blue, and the theoretical isotope distributions are overlaid in red. The close match for each isotope peak compared to its theoretical value, for both mass and relative intensity, supports that the crosslink peptide has been identified correctly. Mass error is given in parts per million (ppm). Additional peaks in the $H_2{}^{18}O$ spectrum are from coeluting peptide species. $H_2{}^{18}O$ labeling validates the presence of a crosslink between two peptides containing residues S341 and C291 in Tau. Created with BioRender.com and Plotly.[34]

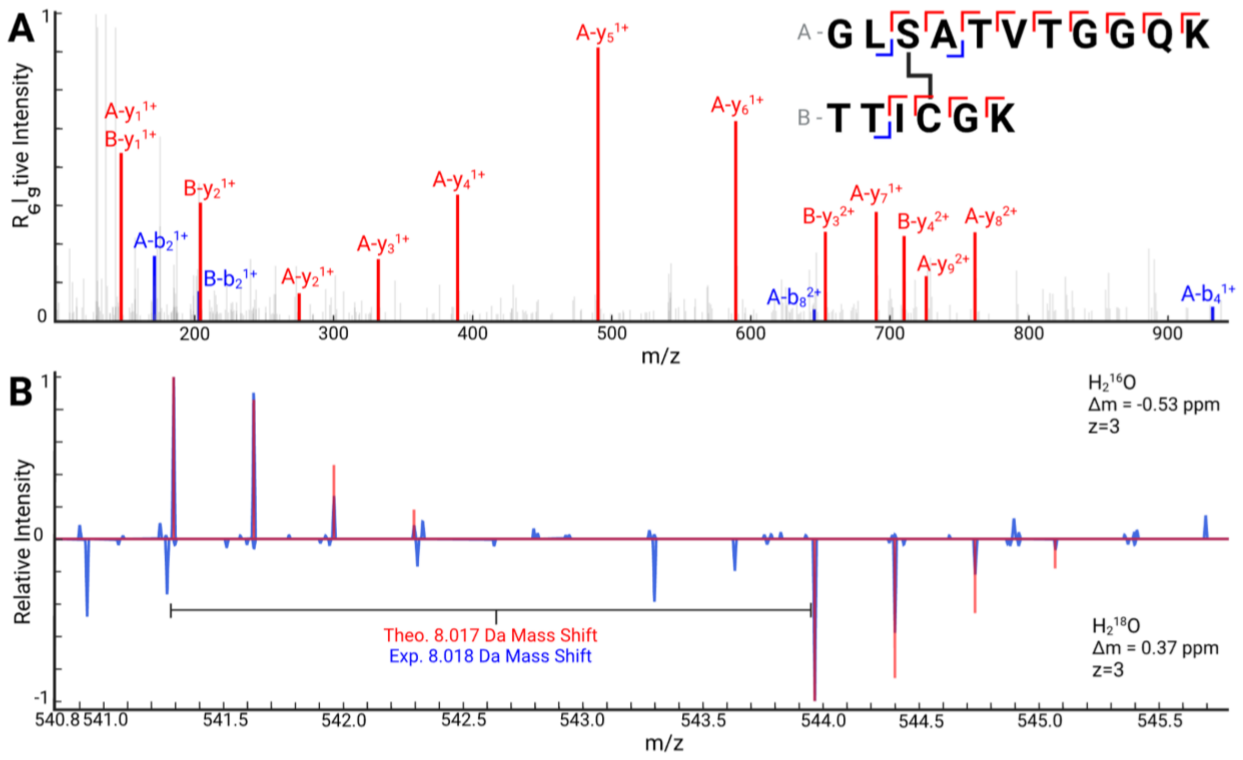

Supplementary Figure 5 Evidence for a DHAA-mediated crosslink between residues S114-C109 in myelin proteolipid protein (PLP1). **(A)** The MS2 fragmentation spectrum for the precursor mass corresponding to the crosslinked species, where m/z represents the mass to charge ratio. It shows thirteen y-ions and three b-ions, including four especially important fragments (A-y_9, A-b_4, B-y_3, and B-y_4) that include the crosslink. Fragmentation throughout the peptide is sufficient to accurately assign the crosslink to S114 and C109. **(B)** A mirror plot is shown with MS1 data derived from $H_2{}^{16}O$ samples on top (usual preparation) and $H_2{}^{18}O$ samples on the bottom (heavy isotope preparation). Raw data are shown in blue, and the theoretical isotope distributions are overlaid in red. The match for each isotope peak compared to its theoretical value, for both mass and relative intensity, supports that the crosslink peptide has been identified correctly. Mass error is given in parts per million (ppm). Additional peaks in the $H_2{}^{18}O$ spectrum are from coeluting peptide species. $H_2{}^{18}O$ labeling validates the presence of a crosslink between two peptides containing residues S114 and C109 in PLP1. Created with BioRender.com and Plotly.[34]

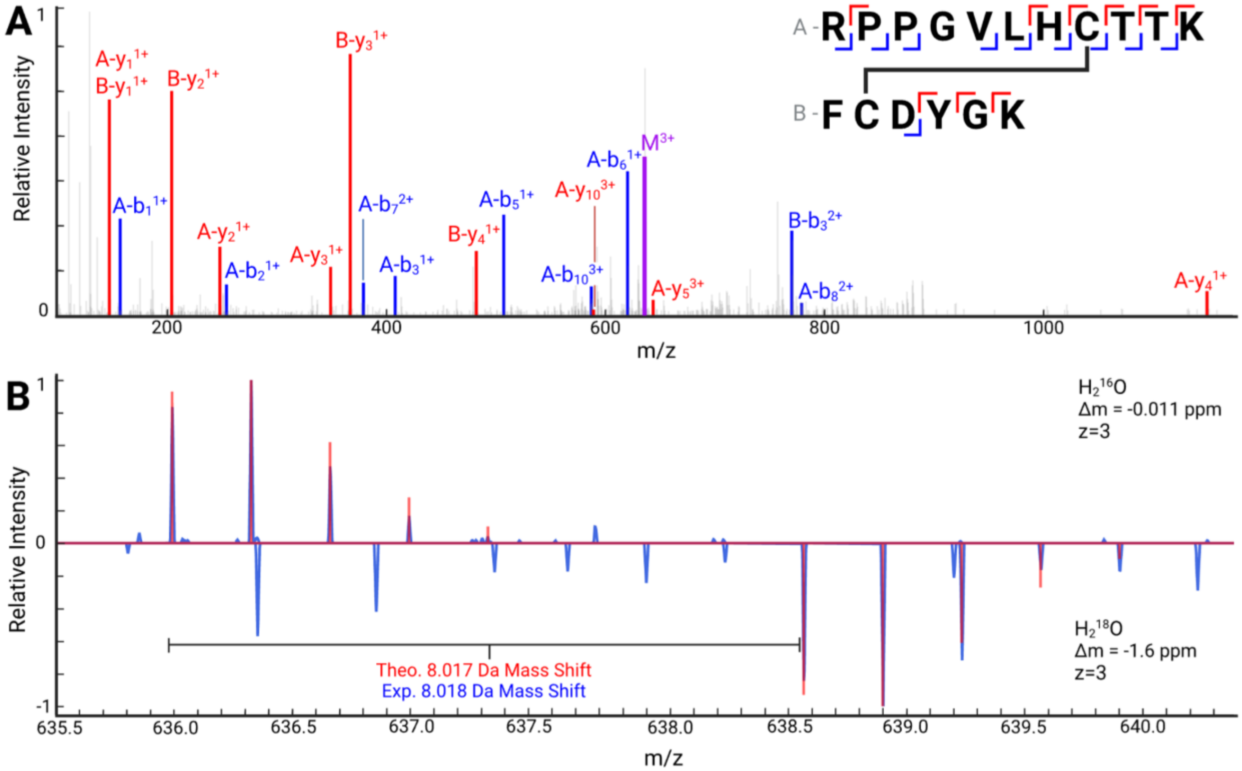

Supplementary Figure 6 Evidence for a DHAA-mediated crosslink between residues C252-C257 in 2,′3′-cyclic nucleotide 3′-phosphodiesterase (CNP). **(A)** The MS2 fragmentation spectrum for the precursor mass corresponding to the crosslinked species, where m/z represents the mass to charge ratio. It shows nine y-ions and ten b-ions, including seven especially important fragments (A-y_4, A-y_5, A-y_{10}, A-b_8, A-b_9, A-b_{10}, and B-b_3) that include the crosslink. A third type of ion (M^{3+}) is present, which is the unfragmented mass of the crosslinked peptides (shown in purple). Fragmentation throughout the peptide is sufficient to accurately assign the crosslink to C252 and C257. **(B)** A mirror plot is shown with MS1 data derived from $H_2{}^{16}O$ samples on top (usual preparation) and $H_2{}^{18}O$ samples on the bottom (heavy isotope preparation). Raw data are shown in blue, and the theoretical isotope distributions are overlaid in red. The close match for each isotope peak compared to its theoretical value, for both mass and relative intensity, supports that the crosslink peptide has been identified correctly. Mass error is given in parts per million (ppm). Additional peaks in the $H_2{}^{18}O$ spectrum are from coeluting peptide species. $H_2{}^{18}O$ labeling validates the presence of a crosslink between two peptides containing residues C252 and C257 in CNP. Created with BioRender.com and Plotly.[34]

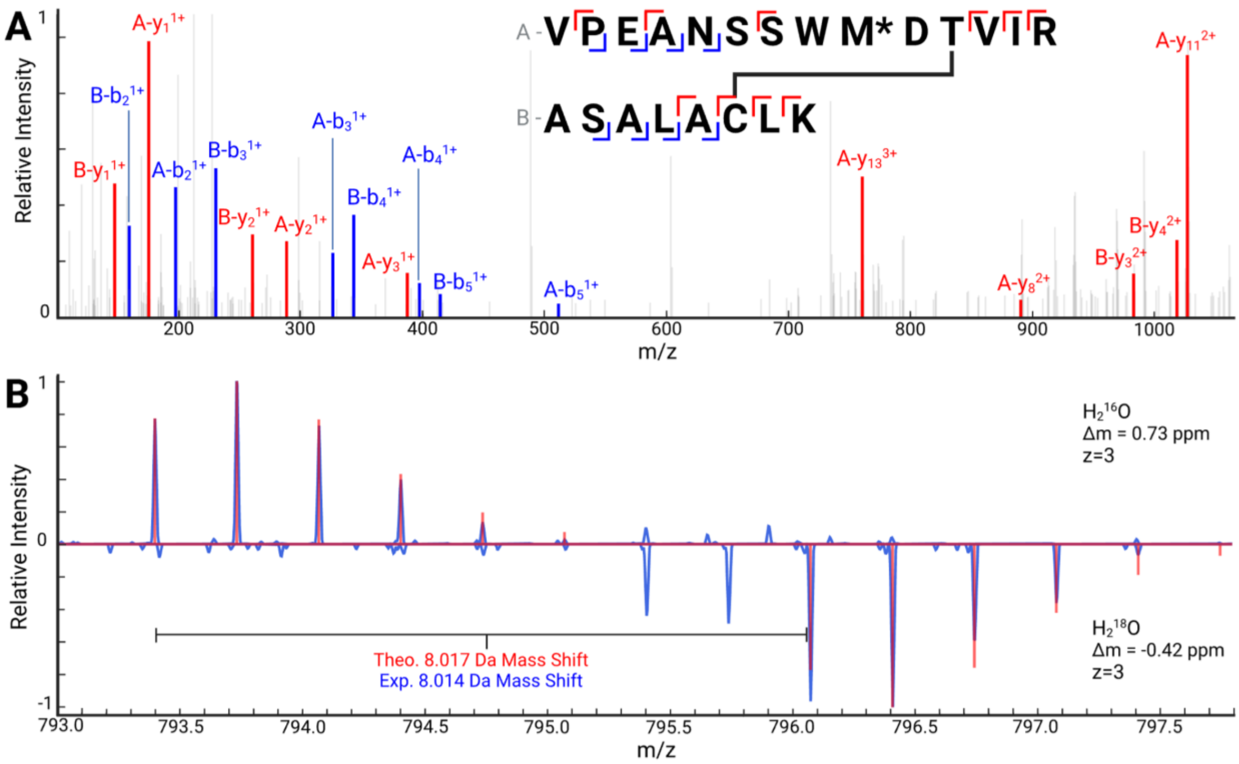

Supplementary Figure 7 Evidence for a DHAA-mediated crosslink between residues S497 or T501 and C488 in dihydrolipoyl transacetylase (DLAT). **(A)** The MS2 fragmentation spectrum for the precursor mass corresponding to the crosslinked species, where m/z represents the mass to charge ratio. "M*" represents that the methionine is oxidized. The MS2 spectrum shows ten y-ions and eight b-ions, including five especially important fragments (A-y_8, A-y_{11}, A-y_{13}, B-y_3, and B-y_4) that include the crosslink. Fragmentation throughout the peptide does not localize the crosslink to a particular DHAA residue, so it is ambiguous whether the crosslink is from S497 or T501 to C488. **(B)** A mirror plot is shown with MS1 data derived from $H_2{}^{16}O$ samples on top (usual preparation) and $H_2{}^{18}O$ samples on the bottom (heavy isotope preparation). Raw data are shown in blue, and the theoretical isotope distributions are overlaid in red. The close match for each isotope peak compared to its theoretical value, for both mass and relative intensity, supports that the crosslink peptide has been identified correctly. Mass error is given in parts per million (ppm). Additional peaks in the $H_2{}^{18}O$ spectrum are from incomplete isotopic labeling of the C-terminal oxygens. $H_2{}^{18}O$ labeling validates the presence of a crosslink between two peptides containing residues S497/T501 and C488 in DLAT. Created with BioRender.com and Plotly.[34]

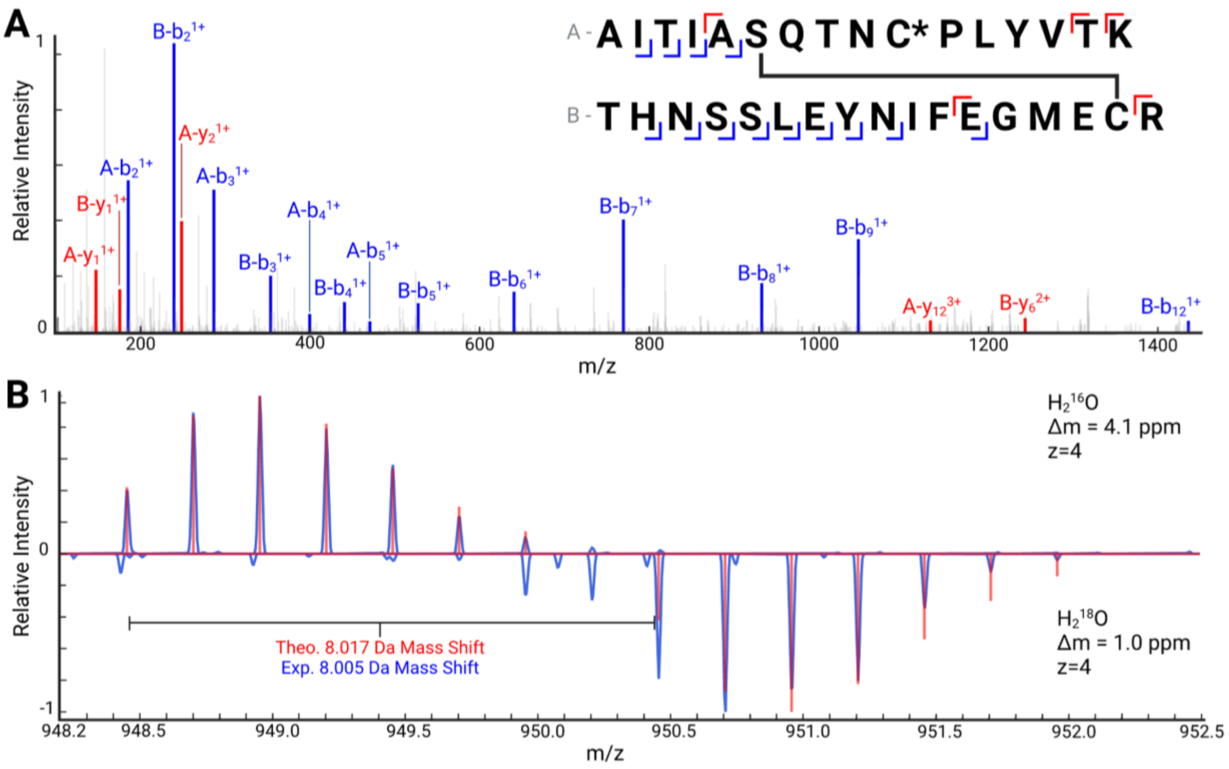

Supplementary Figure 8 Evidence for a DHAA-mediated crosslink between residues S244 or T246 in collapsin response mediator protein 4 (CRMP4) and C439 in collapsin response mediator protein 2 (CRMP2). **(A)** The MS2 fragmentation spectrum for the precursor mass corresponding to the crosslinked species, where m/z represents the mass to charge ratio. "C*" represents that the cysteine is carbamidomethylated. The MS2 spectrum shows five y-ions and thirteen b-ions, including two especially important fragments (A-y_{12} and B-y_6) that include the crosslink. Fragmentation throughout the peptide does not localize the crosslink to a particular DHAA residue, so it is ambiguous whether the crosslink is from S244 or T246 to C439. **(B)** A mirror plot is shown with MS1 data derived from $H_2{}^{16}O$ samples on top (usual preparation) and $H_2{}^{18}O$ samples on the bottom (heavy isotope preparation). Raw data are shown in blue, and the theoretical isotope distributions are overlaid in red. The close match for each isotope peak compared to its theoretical value, for both mass and relative intensity, supports that the crosslink peptide has been identified correctly. Mass error is given in parts per million (ppm). Additional peaks in the $H_2{}^{18}O$ spectrum are from incomplete isotopic labeling of the C-terminal oxygens. $H_2{}^{18}O$ labeling validates the presence of a crosslink between two peptides containing residues S244/T246 in CRMP4 and C439 in CRMP2. Created with BioRender.com and Plotly.[34]

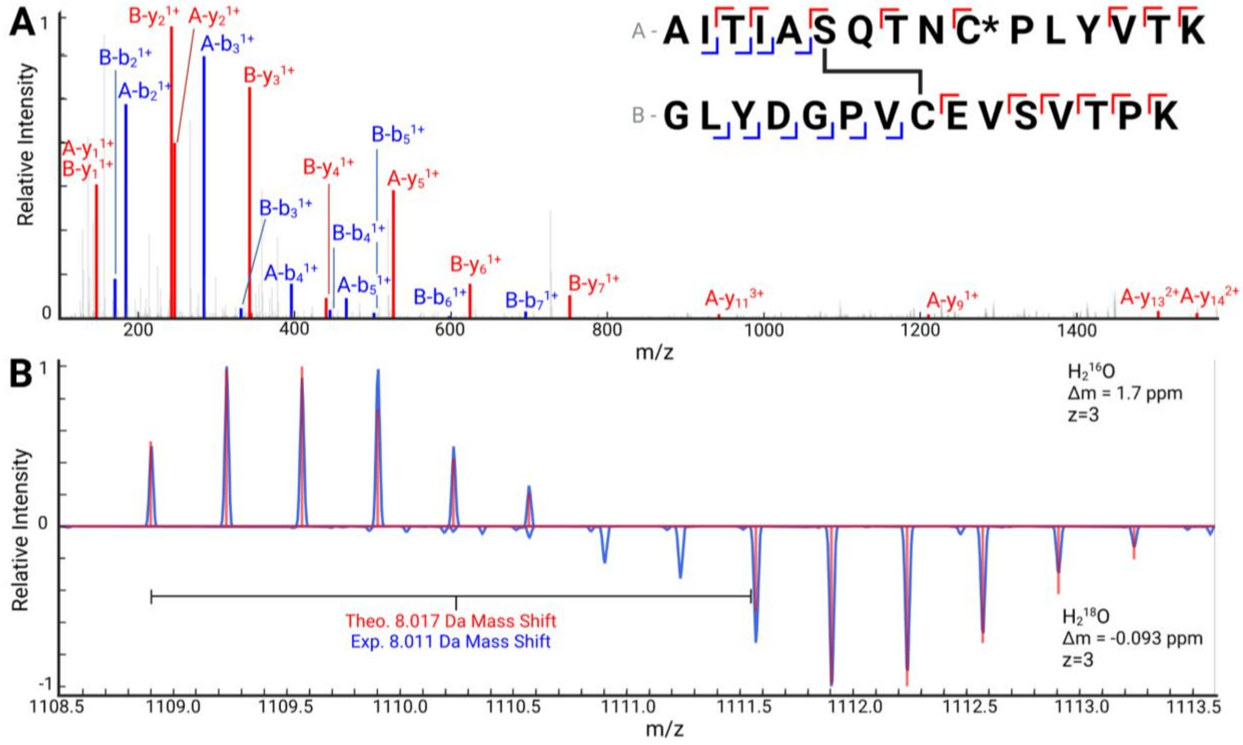

Supplementary Figure 9 Evidence for a DHAA-mediated crosslink between residues S244 in collapsin response mediator protein 4 (CRMP4) and C504 in collapsin response mediator protein 2 (CRMP2). **(A)** The MS2 fragmentation spectrum for the precursor mass corresponding to the crosslinked species, where m/z represents the mass to charge ratio. "C*" represents that the cysteine is carbamidomethylated. The MS2 spectrum shows fourteen y-ions and ten b-ions, including three especially important fragments (A-y_{11}, A-y_{13}, and A-y_{14}) that include the crosslink. Fragmentation throughout the peptide is sufficient to accurately assign the crosslink to S244 in CRMP4 and C504 in CRMP2. **(B)** A mirror plot is shown with MS1 data derived from $H_2{}^{16}O$ samples on top (usual preparation) and $H_2{}^{18}O$ samples on the bottom (heavy isotope preparation). Raw data are shown in blue, and the theoretical isotope distributions are overlaid in red. The close match for each isotope peak compared to its theoretical value, for both mass and relative intensity, supports that the crosslink peptide has been identified correctly. Mass error is given in parts per million (ppm). Additional peaks in the $H_2{}^{18}O$ spectrum are from incomplete isotopic labeling of the C-terminal oxygens. $H_2{}^{18}O$ labeling validates the presence of a crosslink between two peptides containing residues S244 in CRMP4 and C504 in CRMP2. Created with BioRender.com and Plotly.[34]

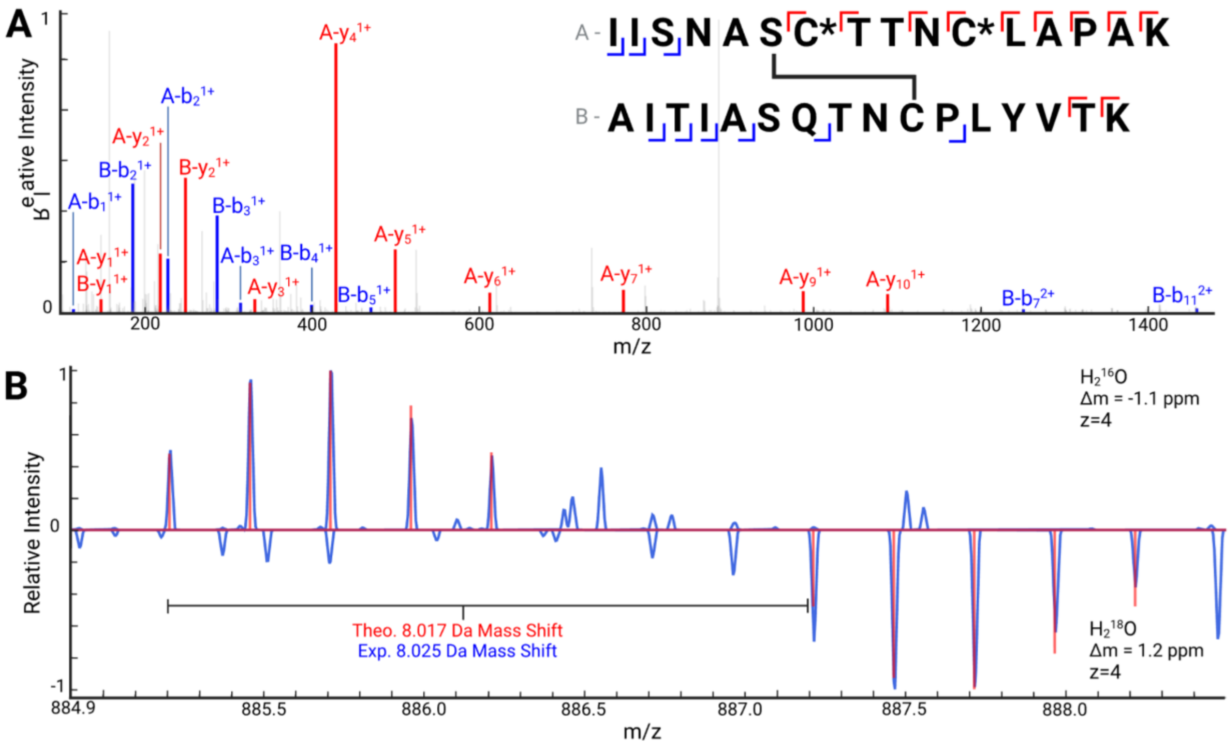

Supplementary Figure 10 Evidence for a DHAA-mediated crosslink between residues S151 in glyceraldehyde-3-phosphate dehydrogenase (GAPDH) and C248 in collapsin response mediator protein 4 (CRMP4). **(A)** The MS2 fragmentation spectrum for the precursor mass corresponding to the crosslinked species, where m/z represents the mass to charge ratio. “C*” represents that the cysteine is carbamidomethylated. The MS2 shows eleven y-ions and nine b-ions, including one especially important fragment (B-b_{11}) that includes the crosslink. Fragmentation throughout the peptide is sufficient to accurately assign the crosslink to S151 in GAPDH and C248 in CRMP4. **(B)** A mirror plot is shown with MS1 data derived from $H_2{}^{16}O$ samples on top (usual preparation) and $H_2{}^{18}O$ samples on the bottom (heavy isotope preparation). Raw data are shown in blue, and the theoretical isotope distributions are overlaid in red. The close match for each isotope peak compared to its theoretical value, for both mass and relative intensity, supports that the crosslink peptide has been identified correctly. Mass error is given in parts per million (ppm). Additional peaks in the $H_2{}^{18}O$ spectrum are from incomplete isotopic labeling of the C-terminal oxygens. $H_2{}^{18}O$ labeling validates the presence of a crosslink between two peptides containing residues S151 in GAPDH and C248 in CRMP4. Created with BioRender.com and Plotly.[34]

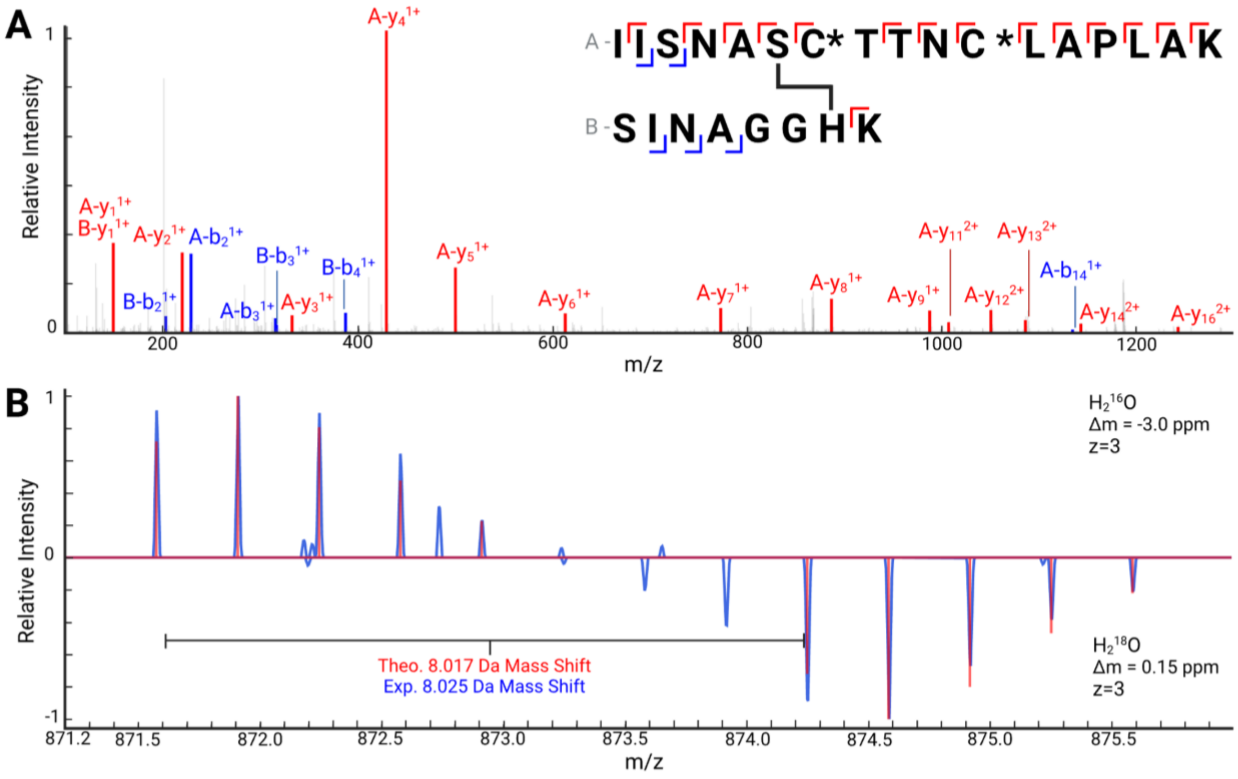

Supplementary Figure 11 Evidence for a DHAA-mediated crosslink between residues S151 in glyceraldehyde-3-phosphate dehydrogenase (GAPDH) and H284 in voltage dependent anion channel 2 (VDAC2). **(A)** The MS2 fragmentation spectrum for the precursor mass corresponding to the crosslinked species, where m/z represents the mass to charge ratio. "C*" represents that the cysteine is carbamidomethylated. The MS2 shows fifteen y-ions and five b-ions, including four especially important fragments (A-y_{12}, A-y_{13}, A-y_{14}, and A-y_{16}) that include the crosslink. Fragmentation throughout the peptide is sufficient to accurately assign the crosslink to S151 in GAPDH and H284 in VDAC2. **(B)** A mirror plot is shown with MS1 data derived from $H_2{}^{16}O$ samples on top (usual preparation) and $H_2{}^{18}O$ samples on the bottom (heavy isotope preparation). Raw data are shown in blue, and the theoretical isotope distributions are overlaid in red. The close match for each isotope peak compared to its theoretical value, for both mass and relative intensity, supports that the crosslink peptide has been identified correctly. Mass error is given in parts per million (ppm). Additional peaks in the $H_2{}^{18}O$ spectrum are from incomplete isotopic labeling of the C-terminal oxygens. $H_2{}^{18}O$ labeling validates the presence of a crosslink between two peptides containing residues S151 in GAPDH and H284 in VDAC2. Created with BioRender.com and Plotly.[34]

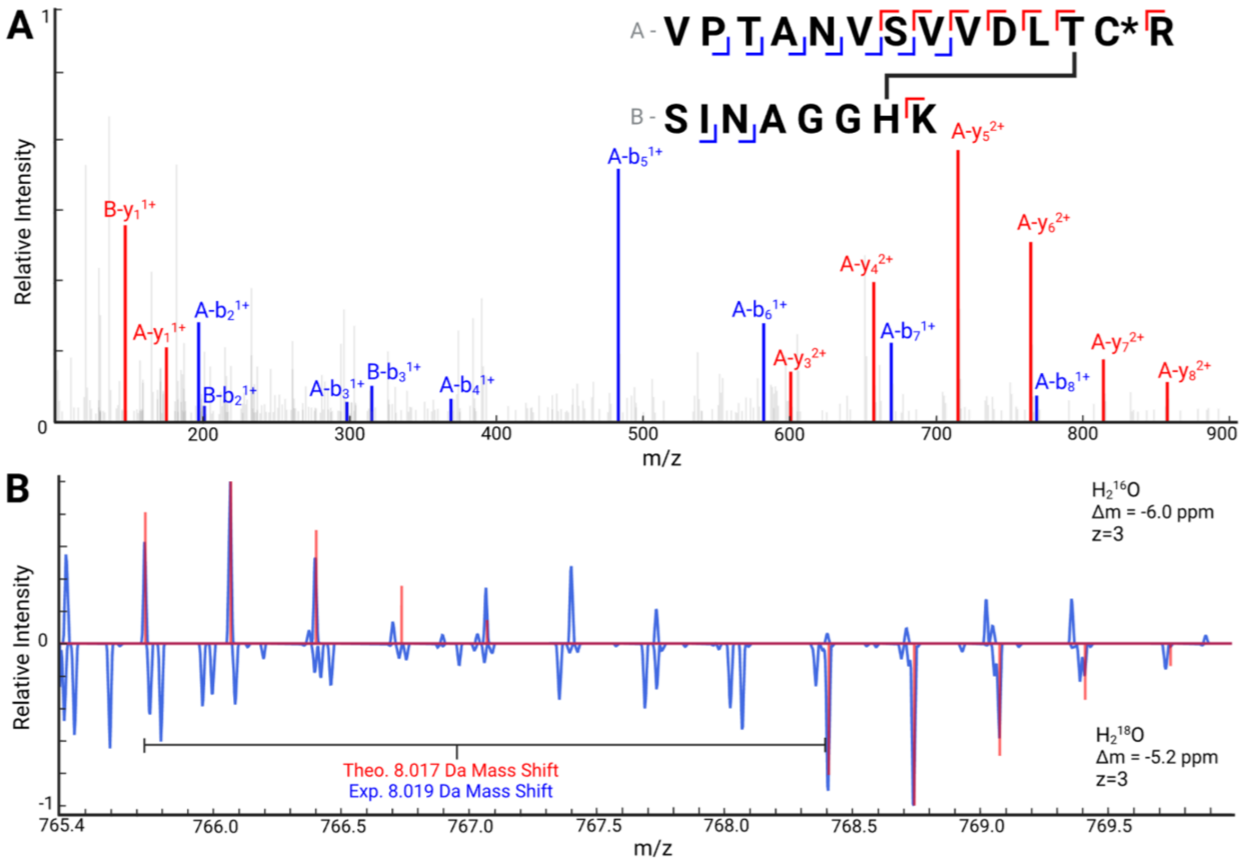

Supplementary Figure 12 Evidence for a DHAA-mediated crosslink between residues T246 in glyceraldehyde-3-phosphate dehydrogenase (GAPDH) and H284 in voltage dependent anion channel 2 (VDAC2). **(A)** The MS2 fragmentation spectrum for the precursor mass corresponding to the crosslinked species, where m/z represents the mass to charge ratio. "C*" represents that the cysteine is carbamidomethylated. The MS2 shows eight y-ions and nine b-ions, including six especially important fragments (A-y_3, A-y_4, A-y_5, A-y_6, A-y_7, and A-y_8) that include the crosslink. Fragmentation throughout the peptide is sufficient to accurately assign the crosslink to T247 in GAPDH and H284 in VDAC2. **(B)** A mirror plot is shown with MS1 data derived from $H_2{}^{16}O$ samples on top (usual preparation) and $H_2{}^{18}O$ samples on the bottom (heavy isotope preparation). Raw data are shown in blue, and the theoretical isotope distributions are overlaid in red. The match for each isotope peak compared to its theoretical value, for both mass and relative intensity, supports that the crosslink peptide has been identified correctly. Mass error is given in parts per million (ppm). Additional peaks in the $H_2{}^{18}O$ spectrum are from incomplete isotopic labeling of the C-terminal oxygens and coeluting peptide species. $H_2{}^{18}O$ labeling

validates the presence of a crosslink between two peptides containing residues T247 in GAPDH and H284 in VDAC2. Created with BioRender.com and Plotly.[34]

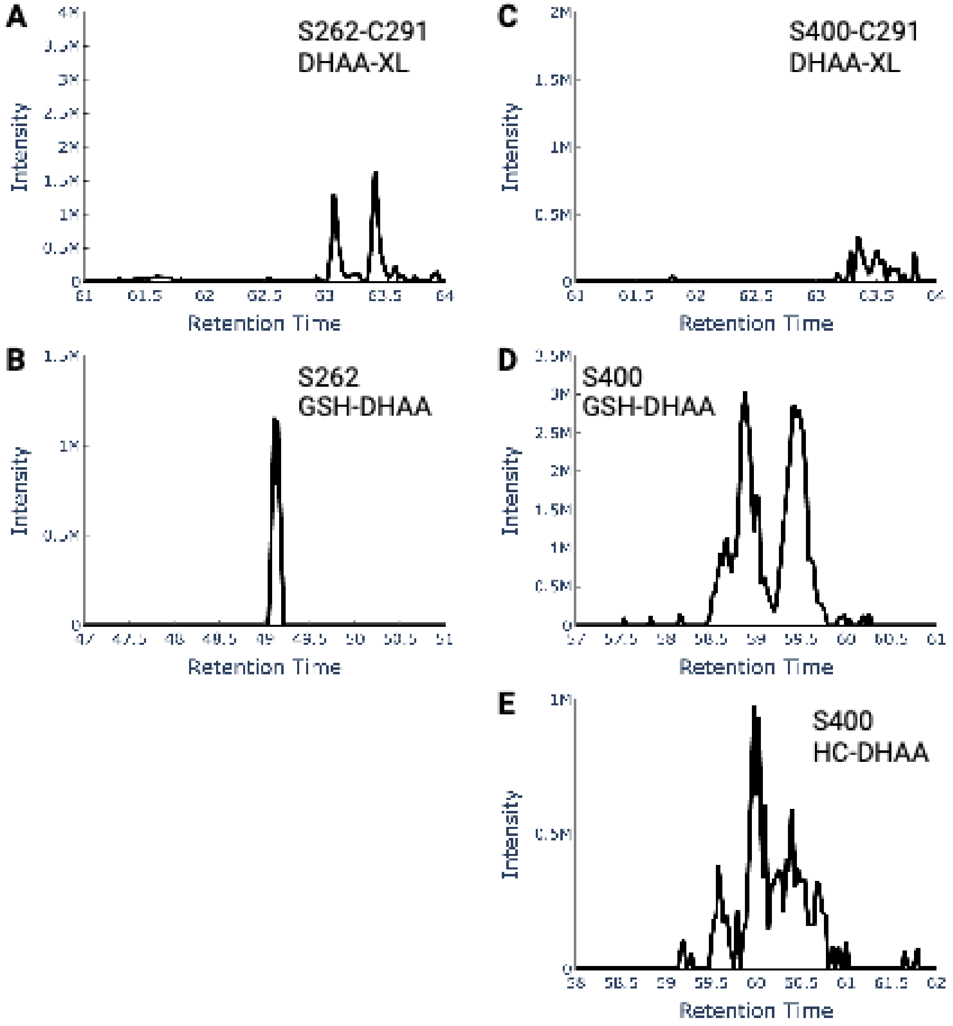

Supplementary Figure 13 Extracted ion chromatograms (XICs) for DHAA-XLs, GSH-DHAAs, and HC-DHAAs from Tau S262 and S400. (**A**) XIC for m/z between 427.46-427.48, representing S262-C291 crosslinked tryptic peptides (IGSTENLK-CGSKDNIK, m=1706.874, z=4). (**B**) XIC for m/z between 604.62-604.64, representing IGS[GSH-DHAA on S]TENLKHQPGGK (m=1810.864, z=3). (**C**) XIC for m/z between 487.49-487.50, representing S400-C291 crosslinked tryptic peptides (SPVVSGDTSPR-CGSKDNIK, m=1946.960, z=4). (**D**) XIC for m/z between 676.57-676.59, representing AKTDHGAEIVYKSPVVS[GSH-DHAA on S]GDTSPR (m=2702.297, z=4). (**E**) XIC for m/z between 644.31-644.33, representing AKTDHGAEIVYKSPVVS[HC-DHAA on S]GDTSPR[Carbamyl on R] (m=2573.255, z=4).

The XICs represented in **A** and **D** are identical-mass chromatographic doublets, which may indicate a racemic mixture. DHAAs are trigonal planar and lose stereochemistry at the α-carbon where they are formed. When the stereocenter is reformed as a result of nucleophilic

addition to the β-carbon and tautomerization, it may form an L- or D-amino acid. If the reaction occurs spontaneously, the products will likely be split evenly between L- and D-isomers, resulting in a racemic mixture. Reverse phase separation on C18 columns can sometime separate these diastereomers, and this is likely the reason that we observe the chromatographic doublets in **A** and **D**. Glutathionylation of DHAAs may be catalyzed by LanCL1/2, and that reaction is known to be stereoselective for the L-isomer.[15] This stereoselective reaction would result in a single GSH-DHAA species, and thus one chromatographic peak, as observed in **B**. However, the reaction may occur spontaneously, and there is also evidence of that expected chromatographic doublet observed in **D**.

There is no known enzyme in the human proteome to catalyze crosslink formation by reaction of DHAAs with nucleophilic protein side chains, so observation of a doublet for the crosslinked species in **A** is reasonable, likely reflective of a spontaneous reaction yielding a racemic mixture. The signal-to-noise in **C** and **E** is insufficient to allow the presence or absence of doublets to be determined. Notably, when the S400-C291crosslink was synthesized *in vitro* and subjected to MS analysis at a high concentration, a single species was observed, rather than a doublet (data not shown). This may indicate that there is something inherent about the S400 site that controls stereochemistry, or it may represent an inability of the reverse phase chromatography employed to separate these diastereomers.

Supplementary Figure 14 Relative occupancy of modifications at each protein residue that has been identified as an eliminylation site. The legend defining colors for the various modifications is shown next to the first plot and pertains to all plots. Occupancy was estimated for each eliminylation site that was (i) identified in the Steen sarkosyl-insoluble datasets; (ii) identified by a DHAA conjugate as a site of eliminylation; and (iii) found in at least 50% of biological replicates.

All data presented here are occupancy estimates based on FlashLFQ normalized intensities. These data are extrapolated from mass spectral intensities and are complex measurements that are ultimately determined by several factors, such as peptide abundance, coeluting species abundance, and ionization efficiencies of each species. The raw data used to assemble these graphs is available in Supplementary Table 4.

Each eliminylation site residue is listed along the x-axis. Relative occupancy fraction (y-axis) is displayed as stacked bars for the various modifications. Data is derived from the sarkosyl-insoluble samples from the Steen dataset for AD specimens (left bar) and age-matched controls (right bar) for each residue. Error bars represent the standard error of the mean. Figures created with Plotly,[34] legend generated with BioRender.com.

Legend

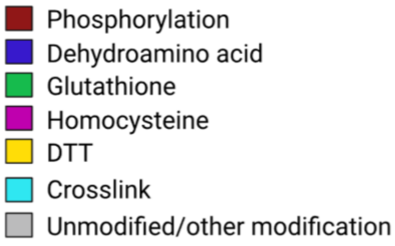

A. Tau Occupancy

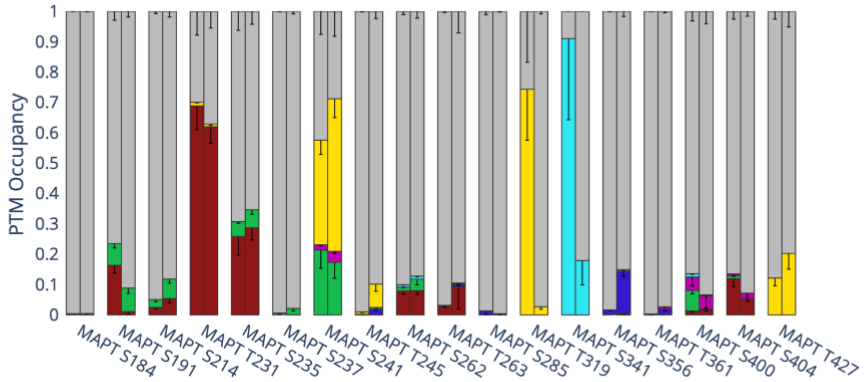

B. CRMP2 Occupancy

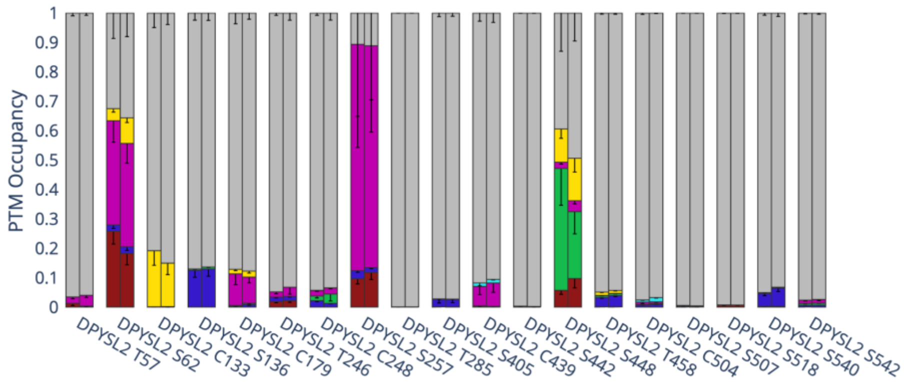

C. GAPDH Occupancy

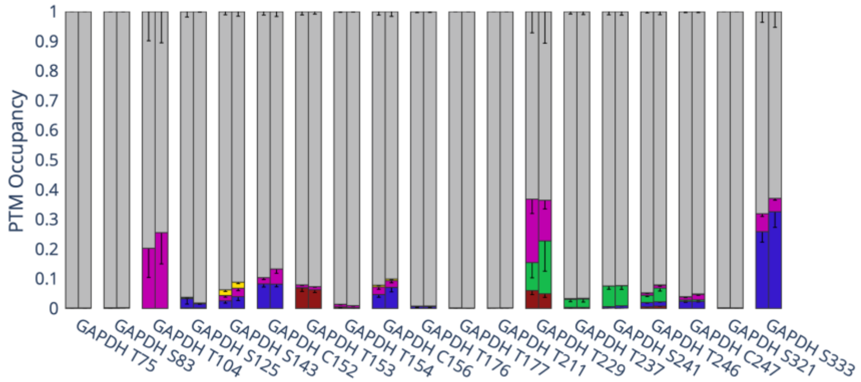

D. PKM Occupancy

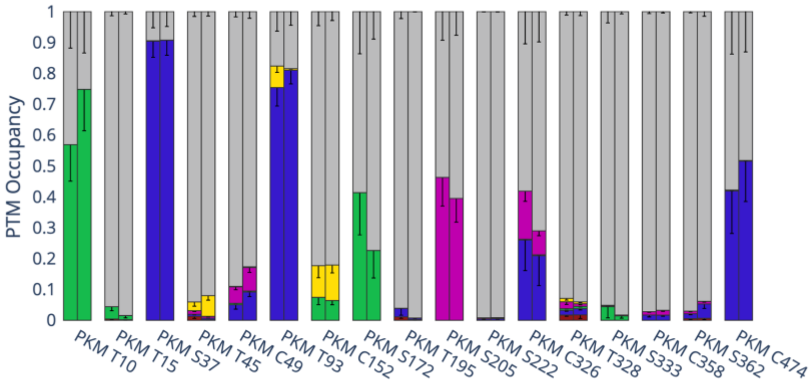

E. VCAN Occupancy

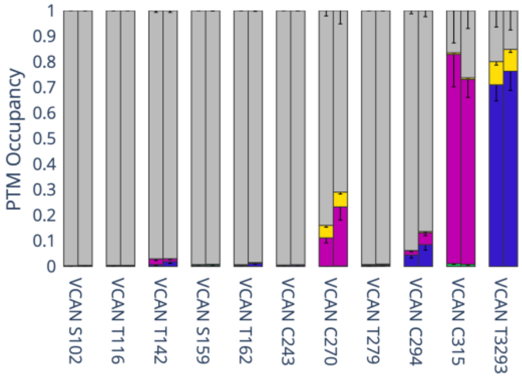

F. All Other Proteins

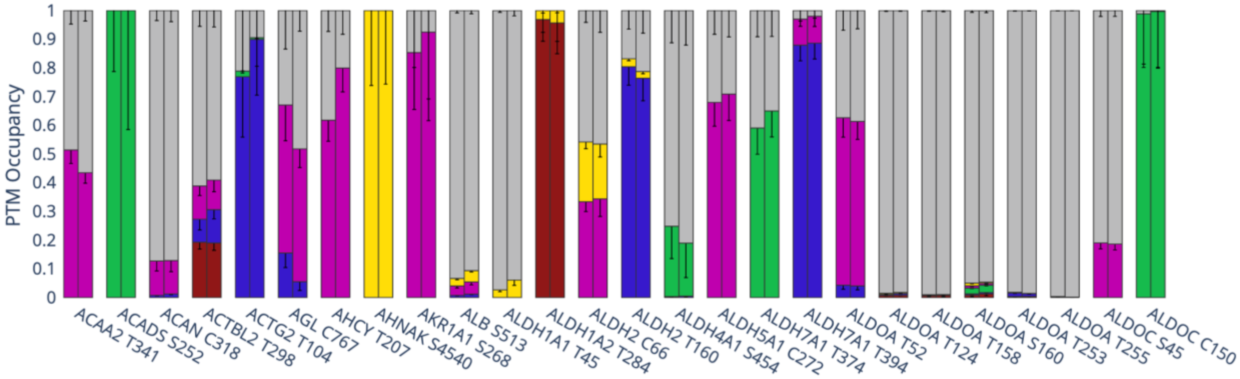

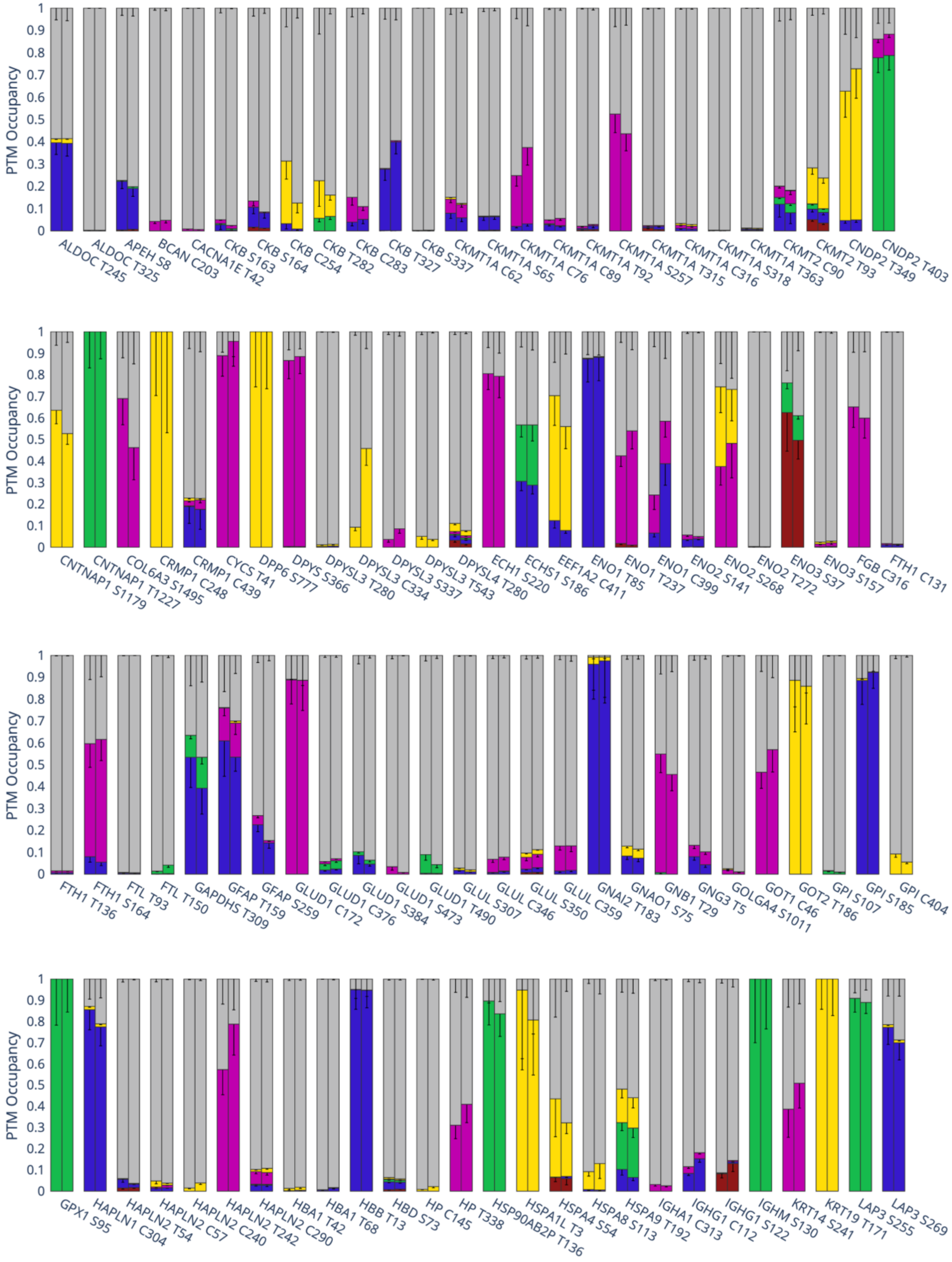
PTM Occupancy
0
0.1
0.2
0.3
0.4
0.5
0.6
0.7
0.8
0.9
1
ALDOC T245
ALDOC T325
APEH S8
BCAN C203
CACNA1E T42
CKB S163
CKB S164
CKB C254
CKB T282
CKB C283
CKB T327
CKB S337
CKMT1A C62
CKMT1A S65
CKMT1A C76
CKMT1A C89
CKMT1A T92
CKMT1A S257
CKMT1A T315
CKMT1A C316
CKMT1A S318
CKMT1A T363
CKMT2 C90
CKMT2 T93
CNDP2 T349
CNDP2 T403
CNTNAP1 S1179
CNTNAP1 T1227
COL6A3 S1495
CRMP1 C248
CRMP1 C439
CYCS T41
DPP6 S777
DPYS S366
DPYSL3 T280
DPYSL3 C334
DPYSL3 S337
DPYSL3 T543
DPYSL4 T280
ECH1 S220
ECHS1 S186
EEF1A2 C411
ENO1 T85
ENO1 T237
ENO1 C399
ENO2 S141
ENO2 S268
ENO2 T272
ENO3 S37
ENO3 S157
FGB C316
FTH1 C131
FTH1 T136
FTH1 S164
FTL T93
FTL T150
GAPDHS T309
GFAP T159
GFAP S259
GLUD1 C172
GLUD1 C376
GLUD1 S384
GLUD1 S473
GLUD1 T490
GLUL S307
GLUL C346
GLUL S350
GLUL C359
GNAI2 T183
GNAO1 S75
GNB1 T29
GNG3 T5
GOLGA4 S1011
GOT1 C46
GOT2 T186
GPI S107
GPI S185
GPI C404
GPX1 S95
HAPLN1 C304
HAPLN2 T54
HAPLN2 C57
HAPLN2 C240
HAPLN2 T242
HAPLN2 C290
HBA1 T42
HBA1 T68
HBB T13
HBD S73
HP C145
HP T338
HSP90AB2P T136
HSPA1L T3
HSPA4 S54
HSPA8 S113
HSPA9 T192
IGHA1 C313
IGHG1 C112
IGHG1 S122
IGHM S130
KRT14 S241
KRT19 T171
LAP3 S255
LAP3 S269

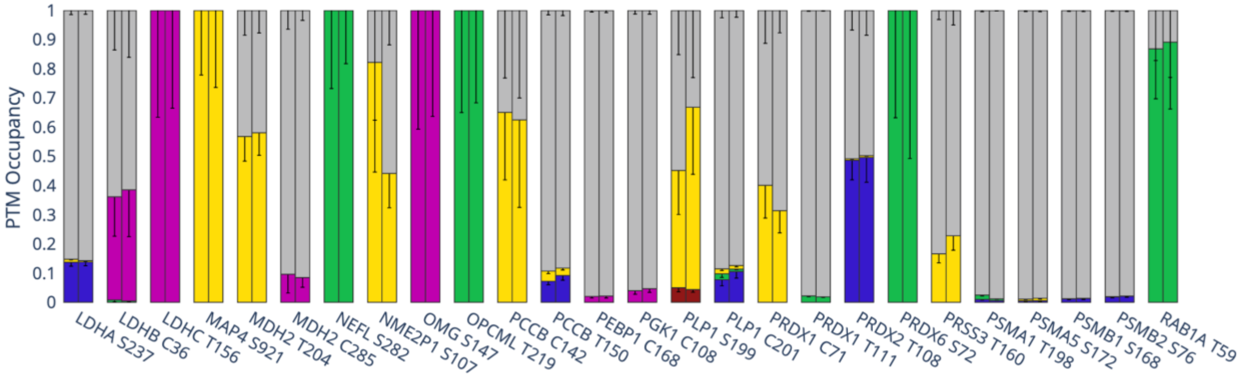
PTM Occupancy
1
0.9
0.8
0.7
0.6
0.5
0.4
0.3
0.2
0.1
0
LDHA S237
LDHB C36
LDHC T156
MAP4 S921
MDH2 T204
MDH2 C285
NEFL S282
NME2P1 S107
OMG S147
OPCML T219
PCCB C142
PCCB T150
PEBP1 C168
PGK1 C108
PLP1 S199
PLP1 C201
PRDX1 C71
PRDX1 T111
PRDX2 T108
PRDX6 S72
PRSS3 T160
PSMA1 T198
PSMA5 S172
PSMB1 S168
PSMB2 S76
RAB1A T59

Supplementary Table 1: Brain specimen details.

Sample	Condition	Age	Sex	Post-mortem interval (hh:mm)
BA339	AD	87	F	3:56
BA359	AD	>90	M	4:40
BA389	AD	76	M	18:03
BA406	AD	85	M	14:16
BA408	AD	80	F	14:05
BA420	AD	89	F	4:15
BA432	AD	87	F	11:45
BA483	AD	>90	F	24:35
BA500	AD	80	M	6:00
BA533	AD	>90	M	23:39
BA130	Control	81	F	19:45
BA186	Control	55	F	4:27
BA452	Control	70	F	5:05

Supplementary Table 2: Collection times (min.) for the eight fractions obtained from offline fractionation of tryptic peptide samples.

	**Pool 1**	**Pool 2**
Fraction 1	0-4	19-20.5
Fraction 2	4-7	20.5-22
Fraction 3	7-10	22-23.5
Fraction 4	10-13	23.5-25
Fraction 5	13-14	25-28
Fraction 6	14-15	28-31
Fraction 7	15-17	31-35
Fraction 8	17-19	35-40

Supplementary Tables 3 and 4 can be found in Supplementary Materials 4 and 5 respectively.

Supplementary Table 5: Numbers of eliminylation sites and proteins containing DHAAs and DHAA conjugates. This table was generated with data exclusively from Wesseling *et al.* (Steen data) to support that DHAAs are an order of magnitude more abundant in sarkosyl-insoluble samples. The comparison in the main text combined identifications made from both Steen and Smith data. This table shows results of a similar comparison of unfractionated samples for both sarkosyl-insoluble and sarkosyl-soluble sample types, yielding a similar order-of-magnitude difference in eliminylation sites.

	**Sarkosyl-Insoluble**		**Sarkosyl-Soluble**	
	Sites	Proteins	Sites	Proteins
Any Identification	424	159	105	55
>50% of Specimens	265	114	59	35
>80% of Specimens	122	59	17	15
100% of Specimens	18	16	2	2

Supplementary Table 6: Crosslinks potentially identified, but not included in the main list of 11 crosslinks (Table 3) due to missing some aspect(s) of strong evidence. "Weak MS2" refers to the MS2 spectra for these species not being strongly supportive (contained insufficient fragments and did not show evidence of fragments with the crosslink). "1% FDR" means the species was not identified in a search against the whole proteome as a modification at the site of interest, as described in "*Final bottom-up proteomics search with crosslink-appended, GPTMD-modified Swiss-Prot database*." in Methods.

Protein	DHA Residue	Protein	Nucleophile	Reason for Rejection
Amyloid-β	S26	Amyloid-β	H6	$H_2{}^{18}O$ labeling did not validate
NEFM	T264	FGG	H426	Weak MS2, 1% FDR
NEFM	S592	CRMP2	K254	Weak MS2, 1% FDR
CRMP4	T246	PLP	C228	Missed Monoisotopic
CRMP4	T246	GAPDH	C152	Weak MS2, 1% FDR
CRMP4	T246	GAPDH	C247	Weak MS2, 1% FDR
ENO3	C399	ENO3	H190|H191	Weak MS2, 1% FDR
ENO3	C357	ENO3	H371	Weak MS2, 1% FDR
ENO2	S370	ENO2	C357	Weak MS2, 1% FDR
Midkine	C94	Midkine	C126	1% FDR search

Supplementary Table 7 and 8 can be found in Supplementary Materials 6 and 7 respectively.

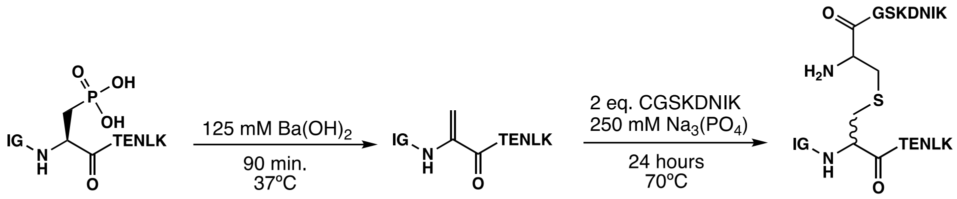

Supplementary Scheme 1: Eliminylation and subsequent crosslinking reaction to generate the S262-C291 synthetic standard from model peptides.

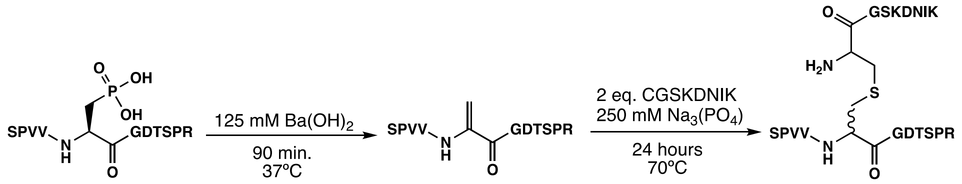

Supplementary Scheme 2: Eliminylation and subsequent crosslinking reaction to generate the S400-C291 synthetic standard from model peptides.

Supplementary References

1. Wesseling H, Mair W, Kumar M, et al. Tau PTM Profiles Identify Patient Heterogeneity and Stages of Alzheimer's Disease. *Cell*. 2020;183(6):1699-1713.e13. doi:10.1016/j.cell.2020.10.029

2. Forman HJ, Zhang H, Rinna A. Glutathione: Overview of its protective roles, measurement, and biosynthesis. *Molecular Aspects of Medicine*. 2009;30(1-2):1-12. doi:10.1016/j.mam.2008.08.006

3. Lai KY, Galan SRG, Zeng Y, et al. LanCLs add glutathione to dehydroamino acids generated at phosphorylated sites in the proteome. *Cell*. 2021;184(10):2680-2695.e26. doi:10.1016/j.cell.2021.04.001

4. Rajagopalan P, Hua X, Toga AW, Jack CR, Weiner MW, Thompson PM. Homocysteine effects on brain volumes mapped in 732 elderly individuals. *NeuroReport*. 2011;22(8):391-395. doi:10.1097/WNR.0b013e328346bf85

5. Purves D, Augustine G, Fitzpatrick D. Neuroscience. In: 2nd Edition. Sinauer Associates; 2001.

6. Brennan DF, Barford D. Eliminylation: a post-translational modification catalyzed by phosphothreonine lyases. *Trends in Biochemical Sciences*. 2009;34(3):108-114. doi:10.1016/j.tibs.2008.11.005

7. Griebel G, Stemmelin J, Lopez-Grancha M, et al. The selective GSK3 inhibitor, SAR502250, displays neuroprotective activity and attenuates behavioral impairments in models of neuropsychiatric symptoms of Alzheimer's disease in rodents. *Sci Rep*. 2019;9(1):18045. doi:10.1038/s41598-019-54557-5

8. Umfress A, Singh S, Ryan KJ, et al. Systemic Administration of a Brain Permeable Cdk5 Inhibitor Alters Neurobehavior. *Front Pharmacol*. 2022;13:863762. doi:10.3389/fphar.2022.863762

9. Jun J, Moon H, Yang S, et al. Carbamate JNK3 Inhibitors Show Promise as Effective Treatments for Alzheimer's Disease: In Vivo Studies on Mouse Models. *J Med Chem*. 2023;66(9):6372-6390. doi:10.1021/acs.jmedchem.3c00393

10. Chun YS, Kim MY, Lee SY, et al. MEK1/2 inhibition rescues neurodegeneration by TFEB-mediated activation of autophagic lysosomal function in a model of Alzheimer's Disease. *Mol Psychiatry*. 2022;27(11):4770-4780. doi:10.1038/s41380-022-01713-5

11. Khater S, Mohanty D. Genome-Wide Search for Eliminylating Domains Reveals Novel Function for BLES03-Like Proteins. *Genome Biology and Evolution*. 2014;6(8):2017-2033. doi:10.1093/gbe/evu161

12. Dong SH, Tang W, Lukk T, Yu Y, Nair SK, Van Der Donk WA. The enterococcal cytolysin synthetase has an unanticipated lipid kinase fold. *eLife*. 2015;4:e07607. doi:10.7554/eLife.07607

13. Mandal PK, Shukla D, Tripathi M, Ersland L. Cognitive Improvement with Glutathione Supplement in Alzheimer's Disease: A Way Forward. *JAD*. 2019;68(2):531-535. doi:10.3233/JAD-181054

14. Vincent I, Zheng JH, Dickson DW, Kress Y, Davies P. Mitotic phosphoepitopes precede paired helical filaments in Alzheimer's disease. *Neurobiology of Aging*. 1998;19(4):287-296. doi:10.1016/S0197-4580(98)00071-2

15. Weaver C. Conformational change as one of the earliest alterations of tau in Alzheimer's disease. *Neurobiology of Aging*. 2000;21(5):719-727. doi:10.1016/S0197-4580(00)00157-3

16. Cripps D, Thomas SN, Jeng Y, Yang F, Davies P, Yang AJ. Alzheimer Disease-specific Conformation of Hyperphosphorylated Paired Helical Filament-Tau Is Polyubiquitinated through Lys-48, Lys-11, and Lys-6 Ubiquitin Conjugation. *Journal of Biological Chemistry*. 2006;281(16):10825-10838. doi:10.1074/jbc.M512786200

17. Cowan CM, Mudher A. Are Tau Aggregates Toxic or Protective in Tauopathies? *Front Neurol*. 2013;4. doi:10.3389/fneur.2013.00114

18. Ghag G, Bhatt N, Cantu DV, et al. Soluble tau aggregates, not large fibrils, are the toxic species that display seeding and cross-seeding behavior. *Protein Science*. 2018;27(11):1901-1909. doi:10.1002/pro.3499

19. Jicha GA, Donnell AO, Weaver C, Angeletti R, Davies P. Hierarchical Phosphorylation of Recombinant Tau by the Paired-Helical Filament-Associated Protein Kinase Is Dependent on Cyclic AMP-Dependent Protein Kinase. *Journal of Neurochemistry*. 1999;72(1):214-224. doi:10.1046/j.1471-4159.1999.0720214.x
