# Supplementary material for: Dehydroamino acids and their crosslinks in Alzheimer’s disease aggregates
Source: Brain Commun. 2025 Jan 16;7(1):fcaf019. doi: 10.1093/braincomms/fcaf019 (PMC11775630; doi:10.1093/braincomms/fcaf019)
Supplement: fcaf019_Supplementary_Data [file fcaf019_supplementary_data.zip › Supplementary Scripts.pdf]


Generate cite specific data from MetaMorpheus output (C# script)

```
[Test]
public static void Junk7()
{
    string fileLabel = "SteenDiscovery";

    Dictionary<(string, string), string>
baseSequencefullSequencQvaluePepQvalueforPSMs = new
Dictionary<(string, string), string>();

    using (StreamReader sr = new StreamReader(@"AllPSMs" + fileLabel
+ ".psmtsv"))
    {
        bool continueReading = true;
        string line;
        while ((line = sr.ReadLine()) != null && continueReading)
        {
            string[] fields = line.Split('\t');
            string baseSequence = fields[12];
            string fullSequence = fields[13];
            string targetDecoyContam = fields[38];
            string qValue = fields[50];
            string pepQValue = fields[55];
            if (targetDecoyContam == "T" && double.TryParse(qValue,
out double qValueDouble))
            {
                if (qValueDouble < 0.01 && !
baseSequencefullSequencQvaluePepQvalueforPSMs.ContainsKey((baseSeque
nce, fullSequence)))
                {

baseSequencefullSequencQvaluePepQvalueforPSMs.Add((baseSequence,
fullSequence), qValue + "\t" + pepQValue);
                }

            }

            if (double.TryParse(qValue, out double qValueDouble2))
            {
                if (qValueDouble2 > 0.01)
                {
                    continueReading = false;
                }
            }
        }
    }

    string psmFilePath =
        @"AllPeptides" + fileLabel + ".psmtsv";
```

```
    List<PsmFromTsv> parsedPeptides =
SpectrumMatchTsvReader.ReadPsmTsv(psmFilePath, out var warnings);
    parsedPeptides = parsedPeptides.Where(p =>
p.DecoyContamTarget.Contains("T")).ToList();

    List<string> interestingMods = new List<string> { "S[Common
Biological:Phosphorylation on S]", "T[Common
Biological:Phosphorylation on T]", "S[Less Common:Dehydroalanine on
S]", "C[Less Common:Dehydroalanine on C]",
        "S[Custom:Homocys on S]", "C[Custom:Homocys on C]",
"T[Custom:Homocys on T]", "T[Less Common:Dehydrobutyrine on T]",
"S[Custom:DTT on S]", "C[Custom:DTT on C]", "C[Custom:DTT on T]",
"T[Custom:Glutathione on T]",
        "S[Custom:Glutathione on S]", "C[Custom:Glutathione on C]",
"S[Custom:TCEP on S]", "T[Custom:TCEP on T]", "C[Custom:TCEP on
C]"  };

    Dictionary<(string, int), List<PsmFromTsv>> genePositionPeptides
= new Dictionary<(string, int), List<PsmFromTsv>>();
    Dictionary<(string, int), List<string>> genePositionMod = new
Dictionary<(string, int), List<string>>();
    Dictionary<string, List<PsmFromTsv>>
noInterestingModsPeptideValues = new Dictionary<string,
List<PsmFromTsv>>();

    foreach (var peptide in parsedPeptides)
    {
        if
(baseSequencefullSequencQvaluePepQvalueforPSMs.ContainsKey((peptide.
BaseSeq, peptide.FullSequence)))
        {
            List<string> foundMods = interestingMods.Where(s =>
peptide.FullSequence.Contains(s)).ToList();
            if (foundMods.Any())
            {
                foreach (var mod in foundMods)
                {
                    string sequence = peptide.FullSequence;
                    string firstCharacter = mod.Substring(0, 1);
                    if (firstCharacter == "Y" || firstCharacter ==
"S" || firstCharacter == "T" ||
                        firstCharacter == "C")
                    {
                        sequence = sequence.Replace(mod,
firstCharacter.ToLowerInvariant());
                    }

                    sequence = sequence.Replace("[I]", "");
                    sequence = sequence.Replace("[II]", "");
                    sequence = sequence.Replace("[III]", "");

                    //eliminate the remaning mods
                    while (sequence.Contains("[") &&
sequence.Contains("]"))
```

```
                    {
                        int firstOpenBracket =
sequence.IndexOf('[');
                        int firstCloseBracket =
sequence.IndexOf(']', firstOpenBracket);
                        if (firstCloseBracket != -1)
                        {
                            sequence =
sequence.Remove(firstOpenBracket, firstCloseBracket -
firstOpenBracket + 1);
                        }
                        else
                        {
                            break;
                        }
                    }

                    List<int> lowercaseLetterPositions = new
List<int>();

                    for (int i = 0; i < sequence.Length; i++)
                    {
                        if (char.IsLower(sequence[i]))
                        {
                            lowercaseLetterPositions.Add(i);
                        }
                    }

                    string firstAndLastAminoAcidPositionInProtein =

peptide.StartAndEndResiduesInProtein.Split('|')[0];
                    firstAndLastAminoAcidPositionInProtein =

firstAndLastAminoAcidPositionInProtein.Substring(1,

firstAndLastAminoAcidPositionInProtein.Length - 2);
                    firstAndLastAminoAcidPositionInProtein =

firstAndLastAminoAcidPositionInProtein.Replace(" to ", "\t");
                    int[] startEnd =
firstAndLastAminoAcidPositionInProtein.Split('\t').Select(int.Parse)
                        .ToArray();
                    lowercaseLetterPositions =
lowercaseLetterPositions.Select(s => s + startEnd[0]).ToList();

                    string allGenesInPsm = peptide.GeneName;
                    string firstGene = "";
                    if (allGenesInPsm.Contains("|"))
                    {
                        string[] genes = allGenesInPsm.Split('|');

                        if (genes[0].Contains(":"))
                        {
                            firstGene = genes[0].Split(':')[1];
```

```
                            foreach (int position in
lowercaseLetterPositions)
                            {
                                if
(genePositionPeptides.ContainsKey((firstGene, position)))
                                {
                                    genePositionPeptides[(firstGene,
position)].Add(peptide);
                                    genePositionMod[(firstGene,
position)]
                                        .Add(mod + "\t" +
peptide.QValue + "\t" + peptide.PEP_QValue);

                                }
                                else
                                {

genePositionPeptides.Add((firstGene, position), new List<PsmFromTsv>
{ peptide });
                                    genePositionMod.Add((firstGene,
position),
                                        new List<string> { mod +
"\t" + peptide.QValue + "\t" + peptide.PEP_QValue });
                                }
                            }
                        }
                        else
                        {
                            firstGene = genes[0];
                            if (genes[0].Contains(":"))
                            {
                                firstGene = genes[0].Split(':')[1];
                            }
                            foreach (int position in
lowercaseLetterPositions)
                            {
                                if
(genePositionPeptides.ContainsKey((firstGene, position)))
                                {
                                    genePositionPeptides[(firstGene,
position)].Add(peptide);
                                    genePositionMod[(firstGene,
position)]
                                        .Add(mod + "\t" +
peptide.QValue + "\t" + peptide.PEP_QValue);

                                }
                                else
                                {

genePositionPeptides.Add((firstGene, position), new List<PsmFromTsv>
{ peptide });
                                    genePositionMod.Add((firstGene,
```

```
position),
                                        new List<string> { mod +
"\t" + peptide.QValue + "\t" + peptide.PEP_QValue });
                                }
                            }
                        }
                    }
                    else
                    {
                        firstGene = peptide.GeneName;
                        if (peptide.GeneName.Contains(":"))
                        {
                            firstGene = peptide.GeneName.Split(':')
[1];
                        }

                        foreach (int position in
lowercaseLetterPositions)
                        {
                            if
(genePositionPeptides.ContainsKey((firstGene, position)))
                            {
                                genePositionPeptides[(firstGene,
position)].Add(peptide);
                                genePositionMod[(firstGene,
position)]
                                    .Add(mod + "\t" + peptide.QValue
+ "\t" + peptide.PEP_QValue);

                            }
                            else
                            {
                                genePositionPeptides.Add((firstGene,
position), new List<PsmFromTsv> { peptide });
                                genePositionMod.Add((firstGene,
position),
                                    new List<string> { mod + "\t" +
peptide.QValue + "\t" + peptide.PEP_QValue });
                            }
                        }
                    }
                }
            }
            else
            {
                if
(noInterestingModsPeptideValues.ContainsKey(peptide.BaseSeq))
                {

noInterestingModsPeptideValues[peptide.BaseSeq].Add(peptide);
                }
                else
                {
```

```
noInterestingModsPeptideValues.Add(peptide.BaseSeq, new
List<PsmFromTsv> { peptide });
                }

            }
        }
    }

    List<(string, string)> outList = new List<(string, string)>();
    List<string> myOut = new List<string>();
    myOut.Add("position" + "\t" + "protein accession" + "\t" +
"gene" + "\t" + "base sequence" + "\t" + "full sequence" + "\t" +
"modification" + "\t" + "Peptide Q-value" + "\t" + "Peptide PEP Q-
Value" + "\t" + "PSM Q-value" + "\t" + "PSM PEP Q-Value");

    foreach (var kvp in genePositionPeptides)
    {
        //if (kvp.Value.Count > 1)
        {
            foreach (var psm in kvp.Value)
            {
                int index = kvp.Value.IndexOf(psm);
                if
(baseSequencefullSequencQvaluePepQvalueforPSMs.ContainsKey((psm.Base
Seq, psm.FullSequence)))
                {
                    myOut.Add(kvp.Key.Item2 + "\t" +
psm.ProteinAccession + "\t" + kvp.Key.Item1 + "\t" + psm.BaseSeq +
"\t" + psm.FullSequence + "\t" + genePositionMod[kvp.Key][index] +
"\t" + baseSequencefullSequencQvaluePepQvalueforPSMs[(psm.BaseSeq,
psm.FullSequence)]);
                    outList.Add((psm.FullSequence, kvp.Key.Item2 +
"\t" + psm.ProteinAccession + "\t" + kvp.Key.Item1 + "\t" +
psm.BaseSeq + "\t" + psm.FullSequence + "\t" +
genePositionMod[kvp.Key][index] + "\t" +
baseSequencefullSequencQvaluePepQvalueforPSMs[(psm.BaseSeq,
psm.FullSequence)]));
                    if
(noInterestingModsPeptideValues.ContainsKey(psm.BaseSeq))
                    {
                        foreach (var psm2 in
noInterestingModsPeptideValues[psm.BaseSeq])
                        {
                            myOut.Add(kvp.Key.Item2 + "\t" +
psm2.ProteinAccession + "\t" + kvp.Key.Item1 + "\t" + psm2.BaseSeq +
"\t" + psm2.FullSequence + "\t" + "No Modifications of Interest" +
"\t" + psm2.QValue + "\t" + psm2.PEP_QValue + "\t" +
baseSequencefullSequencQvaluePepQvalueforPSMs[(psm2.BaseSeq,
psm2.FullSequence)]);
                            outList.Add((psm2.FullSequence,
kvp.Key.Item2 + "\t" + psm2.ProteinAccession + "\t" + kvp.Key.Item1
+ "\t" + psm2.BaseSeq + "\t" + psm2.FullSequence + "\t" + "No
Modifications of Interest" + "\t" + psm2.QValue + "\t" +
psm2.PEP_QValue + "\t" +
```

```
baseSequencefullSequencQvaluePepQvalueforPSMs[(psm2.BaseSeq,
psm2.FullSequence)]));
                        }

noInterestingModsPeptideValues.Remove(psm.BaseSeq);
                    }
                }
                else
                {
                    //myOut.Add(kvp.Key.Item2 + "\t" +
psm.ProteinAccession + "\t" + kvp.Key.Item1 + "\t" + psm.BaseSeq +
"\t" + psm.FullSequence + "\t" + genePositionMod[kvp.Key][index]);

                    //if
(noInterestingModsPeptideValues.ContainsKey(psm.BaseSeq))
                    //{
                    //    foreach (var psm2 in
noInterestingModsPeptideValues[psm.BaseSeq])
                    //    {
                    //        string psmQvalues =
baseSequencefullSequencQvaluePepQvalueforPSMs[(psm2.BaseSeq,
psm2.FullSequence)];
                    //        myOut.Add(kvp.Key.Item2 + "\t" +
psm2.ProteinAccession + "\t" + kvp.Key.Item1 + "\t" + psm2.BaseSeq +
"\t" + psm2.FullSequence + "\t" + "No Modifications of Interest" +
"\t" + psm2.QValue + "\t" + psm2.PEP_QValue + "\t" + psmQvalues);

                    //    }
                    //
noInterestingModsPeptideValues.Remove(psm.BaseSeq);
                    //}
                }

            }
        }
    }

    Dictionary<string, string> quantDict = new Dictionary<string,
string>();
    using (StreamReader sr =
           new StreamReader(
               @"QuantifiedPeptidesNormalized" + fileLabel +
".tsv"))
    {
        string line;
        while ((line = sr.ReadLine()) != null)
        {
            string[] fields = line.Split('\t');
            string fullSequence = fields[0];
            if (!quantDict.ContainsKey(fullSequence))
            {
                quantDict.Add(fullSequence, line);
            }
        }
```

```
    }

    List<string> newOutList = new List<string>();
    newOutList.Add(myOut[0] + "\t" + quantDict["Sequence"]);
    foreach (var kvp in outList)
    {
        if (quantDict.ContainsKey(kvp.Item1))
        {
            newOutList.Add(kvp.Item2 + "\t" + quantDict[kvp.Item1]);
        }
        else
        {
            newOutList.Add(kvp.Item2);
        }
    }

    File.WriteAllLines(@"modPeptides" + fileLabel +
"WithPositionAndQuant_new.txt", newOutList);
}
```


Generate MS2 plots (python)

```
import pandas as pd
import plotly.graph_objs as go

# Load the Excel file
file_path = r'S400 MS2.xlsx'
df = pd.read_excel(file_path)

# Create the Plotly figure
fig = go.Figure()

# Define color and opacity settings
color_map = {
    'Blue': {'color': 'blue', 'opacity': 1, 'width': 4},
    'Red': {'color': 'red', 'opacity': 1, 'width': 4},
    'Grey': {'color': 'grey', 'opacity': 0.2, 'width': 2},
}

# Iterate over each group and add vertical lines
for color, group in df.groupby('Color'):
    if color in color_map:
        trace_color = color_map[color]['color']
        trace_opacity = color_map[color]['opacity']
        trace_width = color_map[color]['width']
    else:
        trace_color = 'black'  # Default color
```

```
        trace_opacity = 0.6  # Default opacity
        trace_width = 2  # Default width

    for _, row in group.iterrows():
        fig.add_trace(go.Scatter(
            x=[row['m/z'], row['m/z']],
            y=[0, row['Intensity']],
            mode='lines',
            line=dict(color=trace_color, width=trace_width),
            opacity=trace_opacity,
            name=color
        ))

# Update the layout
fig.update_layout(
    title='Vertical Line Plot from Excel Data',
    xaxis=dict(
        title='m/z',
        showline=True,
        showgrid=True,
        showticklabels=True,
        linecolor='black',
        linewidth=1,
        ticks='inside',
        tickwidth=1,
        ticklen=5,
        tickcolor='black',
        range=[100, 900]  # Set the range for the x-axis
    ),
    yaxis=dict(
        title='Intensity',
        showline=True,
        showgrid=True,
        showticklabels=True,
        linecolor='black',
        linewidth=1,
        ticks='inside',
        tickwidth=1,
        ticklen=5,
        tickcolor='black',
        range=[0, 70000]  # Set the range for the y-axis
    ),
    plot_bgcolor='rgba(255, 255, 255, 0.9)',  # Set the plot
background color
    width=1800,  # Set the width of the plot
    height=600,  # Set the height of the plot
)

# Save the plot as SVG
svg_output_path = r'Tau S400 MS2.svg'
fig.write_image(svg_output_path, format='svg')

--------------------------------------------------------------------
--------------------------------------------------------------------
```

Generate MS1 plots (python)

```
import pandas as pd
import plotly.graph_objs as go

# Step 1: Read data from Excel
df = pd.read_excel(r'Sigmoidal Shape.xlsx')

# Step 2: Create scatter line plot
fig = go.Figure()

# Step 3: Iterate over each group and add traces with the specified
color and opacity
for color, group in df.groupby('Text Color'):  # Assuming 'Text
Color' is the column indicating the color of the text
    if color == 'Black':
        trace_color = 'royalblue'
        trace_opacity = 1
        width: 1 # Full opacity for black lines
    else:
        trace_color = 'red'
        trace_opacity = 0.8  # 50% opacity for blue lines
        width: 1

    fig.add_trace(go.Scatter(
        x=group['m/z'],
        y=group['Intensity'],
        mode='lines',
        name=color,
        line=dict(color=trace_color, width=5),
        opacity=trace_opacity  # Set opacity
    ))

# Step 4: Add x and y axes
fig.update_xaxes(title_text='t', showline=True, linewidth=1,
linecolor='black', mirror=True, ticks='inside', tickwidth=1,
ticklen=5, dtick=500)
fig.update_yaxes(title_text='Aggregated Protein', showline=True,
linewidth=1, linecolor='black', mirror=True, ticks='inside',
tickwidth=1, ticklen=5, dtick=50000, range=[0, 4826])

# Step 5: Update layout
fig.update_layout(title='Scatter Line Plot from Excel Data',
                  plot_bgcolor='rgba(255, 255, 255, 0.9)',  # Set
the opacity value (0.9 for 90% opacity)
                  width=1000,  # Set width
                  height=500)  # Set height

# Step 6: Show the plot
svg_output_path = r'Sigmoid.svg'
fig.write_image(svg_output_path, format='svg')
```


Generate Volcano Plot (python)

```
import pandas as pd
import plotly.graph_objects as go

# Load the Excel file
file_path = r'Volcano Plot.xlsx'
df = pd.read_excel(file_path, sheet_name='Sheet1')

# Strip any leading/trailing whitespace from the 'Plot Color' column
df['Plot Color'] = df['Plot Color'].str.strip()

# Convert to lower case if there are case mismatches
df['Plot Color'] = df['Plot Color'].str.lower()

# Define a dictionary to map the values in "Plot Color" to specific
colors and opacity
color_map = {
    'teal1': {'color': '#2FE7F1', 'opacity': 0.2},
    'red1': {'color': '#901717', 'opacity': 0.2},
    'blue1': {'color': '#3719CC', 'opacity': 0.2},
    'green1': {'color': '#16BB4D', 'opacity': 0.2},
    'yellow1': {'color': '#FFDD07', 'opacity': 0.2},
    'orange1': {'color': '#BF00AE', 'opacity': 0.2},
    'teal2': {'color': '#2FE7F1', 'opacity': 1.0},
    'red2': {'color': '#901717', 'opacity': 1.0},
    'blue2': {'color': '#3719CC', 'opacity': 1.0},
    'green2': {'color': '#16BB4D', 'opacity': 1.0},
    'yellow2': {'color': '#FFDD07', 'opacity': 1.0},
    'orange2': {'color': '#BF00AE', 'opacity': 1.0},
    # Add more mappings as needed
}

# Define custom legend names if needed
custom_legend_names = {
    'teal1': '',
    'red1': '',
    'blue1': '',
    'green1': '',
    'yellow1': '',
    'orange1': '',
    'teal2': 'Crosslink',
    'red2': 'Phosphorylation',
    'blue2': 'DHAA',
    'green2': 'Glutathione',
    'yellow2': 'DTT',
    'orange2': 'Homocysteine',
    # Add more custom names as needed
}

# Initialize the plotly figure
fig = go.Figure()
```

```
# Loop through each group in the DataFrame
for plot_color, group in df.groupby('Plot Color'):
    # Get the color and opacity for the current group
    if plot_color in color_map:
        trace_color = color_map[plot_color]['color']
        trace_opacity = color_map[plot_color]['opacity']
        legend_name = custom_legend_names.get(plot_color,
plot_color)  # Use custom name if available
    else:
        # Default color and opacity if not found in color_map
        trace_color = 'grey'
        trace_opacity = 0.5
        legend_name = plot_color  # Use the plot color as legend
name if not found in custom_legend_names

    # Add the scatter trace for the current group
    fig.add_trace(go.Scatter(
        x=group['Log2FC'],
        y=group['p-value'],
        mode='markers',
        marker=dict(color=trace_color, opacity=trace_opacity,
size=18),
        marker_line=dict(color='black', width=2),
        name=legend_name,  # Use the custom legend name
        text=group.apply(lambda row: f'Residue: {row["Residue"]}
<br>Gene: {row["gene"]}<br>Modification: {row["Modification"]}',
axis=1),
        hoverinfo='text'
    ))

# Update layout to improve the appearance and set background to
white
fig.update_layout(
    title='Modifications Vary by Condition',
    xaxis_title='log2(FC)',
    yaxis_title='-log10(p-value)',
    legend_title_text='Plot Color',
    plot_bgcolor='white',  # Set plot background to white
    paper_bgcolor='white',  # Set overall background to white
    height=1200,  # Set the height of the plot
    width=1000,  # Set the width of the plot
    xaxis=dict(showgrid=False, zeroline=False, showline=False),  #
Disable x-axis gridlines and axis line
    yaxis=dict(showgrid=False, zeroline=False, showline=False)   #
Disable y-axis gridlines and axis line
)

# Show the figure
svg_output_path = r'VolcanoPlot.svg'
fig.write_image(svg_output_path, format='svg')

--------------------------------------------------------------------
--------------------------------------------------------------------
```

Generate Occupancy Plots (python)

```
import pandas as pd
import plotly.graph_objects as go

# Load the Excel file
file_path = r'Occupancy for Plot.xlsx'
df = pd.read_excel(file_path, header=[0, 1], index_col=0)

# Ensure all data columns are numeric
df = df.apply(pd.to_numeric, errors='coerce')

# Calculate y-axis range using only data columns
data_columns = [col for col in df.columns if 'error' not in col[1]]
y_axis_range = [0, df[data_columns].sum().max() * 1.5]

# Create a figure with the right layout
fig = go.Figure(
    layout=go.Layout(
        height=800,
        width=1100,
        barmode="relative",
        yaxis=dict(
            title=dict(
                text="PTM Occupancy",  # Add your y-axis label text
here
                font=dict(size=40),  # Control the font size of the
y-axis label
                standoff=30
            ),
            showticklabels=True,
            showgrid=False,
            range=[-0.01, 1],
            tick0=0,
            dtick=0.1,
            tickfont=dict(size=35),  # Control the size of the y-
axis tick labels
        ),
        xaxis=dict(
            tickfont=dict(size=35),  # Control the font size of the
x-axis tick labels
        ),
        yaxis2=go.layout.YAxis(
            visible=False,
            matches="y",
            overlaying="y",
            anchor="x",
        ),
        font=dict(size=24),  # This controls general font size,
including legend text
        legend=dict(
            x=1.02,
            bgcolor="rgba(255, 255, 255, 0)",
```

```
        ),
        hovermode="x",
        showlegend=False,
        margin=dict(b=0, t=10, l=50, r=10),  # Adjust left margin
for the y-axis label if necessary
        plot_bgcolor='rgba(0,0,0,0)'
    )
)

# Define colors for the product, revenue pairs
colors = {
    "AD": {
        "Phospho AD": "#901717",
        "Dehydro AD": "#3719CC",
        "Glutathione AD": "#16BB4D",
        "Homocys AD": "#BF00AE",
        "DTT AD": "#FFDD07",
        "Crosslink AD": "#2FE7F1",
        "Unmodified AD": "#BBBBBC",
    },
    "Control": {
        "Phospho AD": "#901717",
        "Dehydro AD": "#3719CC",
        "Glutathione AD": "#16BB4D",
        "Homocys AD": "#BF00AE",
        "DTT AD": "#FFDD07",
        "Crosslink AD": "#2FE7F1",
        "Unmodified AD": "#BBBBBC",
    },
}

# Error column mapping
error_min_mapping = {
    "Phospho AD": "Phospho err min AD",
    "Dehydro AD": "Dehydro err min AD",
    "Glutathione AD": "Glutathione err min AD",
    "Homocys AD": "Homocys err min AD",
    "DTT AD": "DTT err min AD",
    "Crosslink AD": "Crosslink err min AD",
    "Unmodified AD": "U err min AD",
}

error_max_mapping = {
    "Phospho AD": "Phospho err max AD",
    "Dehydro AD": "Dehydro err max AD",
    "Glutathione AD": "Glutathione err max AD",
    "Homocys AD": "Homocys err max AD",
    "DTT AD": "DTT err max AD",
    "Crosslink AD": "Crosslink err max AD",
    "Unmodified AD": "U err max AD",
}

# Add the traces
for i, t in enumerate(colors):
```

```
    if t not in df:
        continue  # Skip if the key is not found in df
    for col in df[t].columns:
        if (df.loc[:, (t, col)].isna() | (df.loc[:, (t, col)] ==
0)).all():
            continue
        error_min_col = error_min_mapping.get(col)
        error_max_col = error_max_mapping.get(col)
        if error_min_col and error_max_col:
            fig.add_bar(
                x=df.index,
                y=df.loc[:, (t, col)],
                error_y=dict(
                    type='data',
                    symmetric=False,
                    array=(df.loc[:, (t, error_max_col)] - df.loc[:,
(t, col)]),
                    arrayminus=(df.loc[:, (t, col)] - df.loc[:, (t,
error_min_col)]),
                    color='black'  # Set error bars to black
                ),
                yaxis=f"y{i + 1}",
                offsetgroup=str(i),
                offset=(i - 1) * 1/3,
                width=1/3,
                legendgroup=t,
                legendgrouptitle_text=t,
                name=col,
                marker_color=colors[t][col],
                marker_line=dict(width=2, color="#333"),
                hovertemplate="%{y}<extra></extra>"
            )

svg_output_path = r'Other Occupancy.svg'
fig.write_image(svg_output_path, format='svg')
```

Retention Time Alignment (python)

```
import pandas as pd

file1_path = r"4-12-24-Jurkat-IC-8-SC-2-calib_Peptides.psmtsv"

file2_path = r"4-13-24-BA500B-F3-Spike-In-IC-4-SC-1-
calib_Peptides.psmtsv"


f1 = pd.read_csv(file1_path, sep = '\t')
f2 = pd.read_csv(file2_path, sep = '\t')

#f1["Full Sequence"] = f1["Full
```

```
Sequence"].str.replace("[Unimod:Label:18O(2) on X]", "") # Remove
mods in the 18O file
f_merge = pd.merge(f1, f2, how = "inner", on = "Full Sequence",
suffixes=["_18O", "_16O"])
print(f_merge.columns)
f_merge["RtDiff"] = f_merge["Scan Retention Time_18O"] -
f_merge["Scan Retention Time_16O"]
#f_merge_mini = f_merge["Base Sequence", "Scan Retention Time_18O",
"Scan Retention Time_16O", "RtDiff"]

f_merge.to_csv(r"RTDiffSynthVSpikein2-S400.tsv", sep = '\t')
```
